# Unravelling *Artemisia* species genetic variation via DNA barcoding, ISSR and RAPD with the development of eco-specific SCAR markers

**DOI:** 10.1186/s12870-025-07058-9

**Published:** 2025-08-07

**Authors:** Yasmin A. Mahgoub, Hebaalla A. Mahmoud, Nadia A. El-Sebakhy, Ingy I. Abdallah

**Affiliations:** Department of Pharmacognosy, Faculty of Pharmacy, Alexandria University, Alexandria, 21521 Egypt

**Keywords:** *Artemisia annua*, *Artemisia herba-alba*, *Artemisia monosperma*, *Artemisia judaica*, DNA-based markers, Genetic profiling

## Abstract

**Background:**

Genus *Artemisia* is one of the largest and most globally spread genera, comprising more than 500 species known for their phytochemical diversity and therapeutic properties. This necessitates the accurate authentication and differentiation of its species. Traditional morphological, microscopical and metabolic profiling methods are often insufficient for reliable discrimination. The aim of this study is the authentication and assessment of the genetic diversity of wild Egyptian *Artemisia* species; *A. herba-alba*, *A. monosperma*, *A. judaica* and cultivated *A. annua* using a combined molecular approach of DNA barcoding, ISSR, RAPD, and the development of eco-specific SCAR markers.

**Results:**

DNA barcoding targeting both nuclear (ITS2) and plastid (psbA-trnH) spacers revealed that ITS2 is recommended over psbA-trnH as the discriminatory barcode of choice since it accurately identified all species with > 99% identity and phylogenetic clustering with greater genetic distances. ISSR fingerprinting with five primers generated 41 polymorphic bands (100% polymorphism) and displayed genetic diversity among the species. However, the morphologically and chemically similar *A. herba-alba* and *A. judaica* remained partly undifferentiated. Therefore, RAPD profiling was implemented as a complementary technique for better and reliable discrimination. RAPD profiling with 27 primers generated 212 bands (99.5% polymorphic). RAPD primers OPA-10 and OPK-07 showed superior differentiation of the *Artemisia* species, while primers OPG-07, OPB-20, OPS-12 and OPD-15 failed to discriminate between the studied species. The reproducible RAPD banding profiles generated by OPG-02, OPG-04, OPA-09 and OPD-15 primers were targeted for the development of species-specific SCAR markers by isolating, cloning, and sequencing the distinct RAPD bands specific for each species. These putative SCAR markers were assessed and validated confirming the identity of the studied species.

**Conclusions:**

An integrated molecular approach combining ITS2 barcoding, ISSR, RAPD, and RAPD-derived SCAR markers offered a reliable strategy for the authentication and discrimination of *Artemisia* species based on their genetic profiles. It is worth mentioning that this is the first report of eco-specific SCAR markers for the Egyptian *Artemisia* species. The developed SCAR markers allow rapid species identification for quality control of medicinal plants, complementing conventional methods and overcoming their limitations. This provides a reproducible, cost-effective strategy for large-scale authentication of medicinal plants.

**Supplementary Information:**

The online version contains supplementary material available at 10.1186/s12870-025-07058-9.

## Introduction

Genus *Artemisia* is amongst the largest and most globally spread genera of family Asteraceae, comprising more than 500 diverse species. The genus is renowned for its rich phytochemical diversity and wide-ranging therapeutic effects, as well as its long history of use in traditional medicine [1, 2]. *Artemisia annua* L. (commonly referred to in China as sweet wormwood or qinghao) is the most prominent species of the genus, known for its antimalarial artemisinin that remains a key component of combination therapy based on artemisinin which is recommended by the WHO for treatment of malaria. *A. annua* is cultivated globally for its artemisinin content [3, 4]. In Egypt and other North African countries, three notable wild *Artemisia* species, namely *Artemisia herba-alba* Asso, *Artemisia monosperma* Del. and *Artemisia judaica* L. are widely used in traditional medicine for their promising medicinal properties [5–7]. These species share certain morphological similarities, particularly *A. herba-alba* and *A. judaica*, and exhibit both similarities and differences in their metabolic profiles [8–11].

Accurate identification and authentication of plant material are critical for ensuring the quality and efficacy of herbal medicines. Morphological and microscopical identification methods are rapid and cost-effective but are inherently subjective, as many plant species, varieties, and even substitutes or adulterants can closely resemble each other morphologically, leading to misidentification. Consequently, chemical profiling/fingerprinting has emerged and is now widely used for the evaluation of plant material. However, metabolomic approaches also have limitations due to the complexity of plant constituents and variability caused by external factors such as cultivation practices, drying, and storage conditions [12–14]. Hence, there is a need for complementary or alternative approaches to metabolic profiling. The rapid advancement of high-throughput sequencing technologies has paved the way for genomics-based techniques for the identification, differentiation, and genetic fingerprinting of plants. Various DNA-based techniques are now employed for genetic profiling and authentication of plant material, including polymerase chain reaction (PCR)-based methods for developing DNA markers such as DNA barcoding, Random Amplified Polymorphic DNA (RAPD), Inter-Simple Sequence Repeats (ISSR), and Sequence Characterized Amplified Region (SCAR) markers. Over the past few decades, DNA markers have proven valuable for authenticating medicinally important plant species and distinguishing morphologically and/or phytochemically similar species or varieties. Each DNA-based marker type has its own benefits and drawbacks, and none is universally ideal [15–17]. Thus, the selection of marker is usually a compromise that is contingent upon several factors such as cost, technical expertise, the specific research objectives, etc. The development of genetic markers undoubtedly offers a reliable method for authentication and differentiation of plants that complements or substitutes morphological, microscopical, and phytochemical profiling. In this context, the inherent variability of their metabolic profiles has driven the application of genetic profiling as a robust complementary technique for the authentication and differentiation of *Artemisia* species.

The aim of this research is the accurate authentication of the four studied *Artemisia* species; *A. annua*, *A. herba-alba*, *A. monosperma* and *A. judaica* through DNA barcoding. Additionally, this study investigates their genetic diversity through ISSR and RAPD techniques, and develops ecotype-specific SCAR markers for the reliable verification and discrimination of these *Artemisia* species.

## Materials and methods

### Plant material

The aerial parts of the three Egyptian *Artemisia* species; *Artemisia herba-alba* Asso, *Artemisia monosperma* Del. and *Artemisia judaica* L. were collected from Makki Village (kilo 90 northern coast road), El Omayed reservation (kilo 83 northern coast road) and Red Sea Governorate, respectively, in May of 2021 and 2022. Additionally, the aerial parts of the cultivated *Artemisia annua* L. were obtained from the experimental garden of the Faculty of Pharmacy, Cairo University, Giza, in May of 2021 and 2022. Representative specimens were kindly identified by Prof. Amal Mohamed Fakhry (Professor of Plant Ecology, Department of Botany and Microbiology, Faculty of Science, Alexandria University). Voucher specimens for the four studied species, *A. annua*, *A. herba-alba*, *A. monosperma* and *A. judaica* were deposited in the herbarium of the Pharmacognosy Department, Faculty of Pharmacy, Alexandria University under the following deposition numbers: AA01, AA02 and AA03; AH01, AH02 and AH03; AM01, AM02 and AM03; AJ01, AJ02 and AJ03, respectively. Following collection, several samples of the freshly collected young leaves of the four *Artemisia* species, free of any pathogenic symptoms, were separately ground in liquid nitrogen using a mortar and pestle and stored at −80℃ for DNA isolation.

### DNA extraction and quantitation

Total genomic DNA was extracted using the i-genomic kit for DNA extraction (Intron biotechnology, Korea) according to the instructions in manufacturer’s manual. DNA concentrations were determined by UV absorbance using a NanoDrop Lite Plus spectrophotometer™ (Thermo Scientific, USA). Purity of the extracted DNA was confirmed by calculating the A260/A280 ratio to verify the absence of protein contamination.

### Molecular techniques

The four studied *Artemisia* species were authenticated via DNA barcoding. ISSR and RAPD profiling were then performed to assess their genetic diversity, and putative RAPD-based SCAR markers were subsequently developed.

### DNA barcoding

The nuclear (ITS2) and the plastid intergenic (psbA-trnH) spacers were targeted for species authentication. The targeted spacers were amplified using corresponding universal primers, that were chosen based on their selectivity and specificity within the Plantae kingdom. Each reaction mixture contained 30 ng DNA template of the four various *Artemisia* species, 1.5 µl forward and reverse primers for each gene, 25 µl Mytaq™ HS Redmix, 2x (Bioline reagent Ltd., UK), and nuclease-free water to a final volume of 50 µl. The annealing temperature was varied over the range of 53–59 ℃ to optimize PCR cycling parameters for high quality, specific PCR products. The primers used and optimal PCR conditions are detailed in Table 1.

**Table 1:** Universal selected primers and optimized PCR conditions for the targeted DNA barcodes

Target gene	Primer name (reference)	Primer Role	Primer sequence (5’ to 3’)	Optimized PCR conditions
ITS2	ITSU3 [18]	Forward primer	CAWCGATGAAGAACGYAGC	95 ℃ 3 min; 35* 95 ℃ 15 s, 55 ℃ 15 s, 72 ℃ 15 s; 72 ℃ 8 min.
	ITSU4 [18]	Reverse primer	RGTTTCTTTTCCTCCGCTTA	
psbA-trnH	PsbA3_F [19]	Forward primer	GTTATGCATGAACGTAATCGTAATGCTC	95 ℃ 3 min; 35* 95 ℃ 15 s, 56 ℃ 15 s, 72 ℃ 15 s; 72 ℃ 8 min.
	trnHf [19]	Reverse primer	CGCGCATGGTGGATTCACAATCC	

The resultant PCR products were analysed and separated by electrophoresis on 1.8% agarose in 1x TAE buffer. Target bands were purified using the GeneJET Gel Extraction Kit (Thermo Scientific co., USA) based on the producer’s guidelines. Purified amplicons were bidirectionally sequenced using the Sanger method with a SeqStudio Flex ™ Series Genetic Analyzer (Thermo Scientific, USA) at Colors Medical Lab., Cairo, Egypt.

### ISSR fingerprinting

Twenty ISSR primers were initially screened, of which five produced clear and reproducible banding patterns suitable for genetic profiling of the *Artemisia* species. PCR amplification was carried out in SENSOQUEST thermal cycler (Germany) according to the following cycling parameters: initial denaturation at 95 ℃ for 3 min; 40 cycles of denaturation at 95 °C for 15 s, annealing at the primer-specific temperature for 15 s, and extension at 72 °C for 15 s; followed by a final extension at 72 °C for 8 min. Annealing temperatures for primers SR-14, SR-16, SR-33, SR-36, and SR-37 were 45.4, 46.6, 53.2, 46, and 51.8 °C, respectively. ISSR primer data are listed in Table 2. The amplicons were resolved on 1.5% agarose gels in 1x TAE buffer at 85 V for 45 min. The gels were stained with ethidium bromide (0.5 µg/mL) added to the cooled agarose solution before pouring the gel and visualized under a UV transilluminator after electrophoresis. The size of each band was estimated using a 100 bp DNA ladder (Biohelix, Taiwan) which was used as a standard DNA marker. All experiments were performed in triplicates for three populations of each species to ensure reproducibility.

**Table 2:** Primer data and analysis of banding profiles obtained using ISSR primers

Primer code	Primer sequence	Total bands	Monomorphic bands	Polymorphic bands	% Polymorphism	PIC	EMR	MI	RP
SR 14	5’-GAGAGAGAGAGAGAYG-3’	7	0	7	100%	0.4464	7	3.1248	5.5
SR 16	5’-GAGAGAGAGAGAGAGAC-3’	10	0	10	100%	0.4	10	4	6
SR 33	5’-ACACACACACACACACCG-3’	8	0	8	100%	0.4219	8	3.3752	5.5
SR 36	5’-AGAGAGAGAGAGAGAGT-3’	10	0	10	100%	0.4375	10	4.375	8.5
SR 37	5’-ACACACACACACACACC-3’	6	0	6	100%	0.4167	6	2.5	4

### RAPD profiling

Fifty-five universal RAPD primers were used for a preliminary screening, of which 27 yielded stable, reproducible banding patterns suitable for RAPD profiling of the *Artemisia* species. Details of the RAPD primers are provided in Table 3. RAPD-PCR reactions (25 µl) contained 30 ng genomic DNA, 12.5 µl MyTaq™ HS Red Mix, 1.5 µl primer, and nuclease-free water to volume. Amplification was carried out in SENSOQUEST thermal cycler (Germany) using the following setup: initial denaturation at 95 ℃ for 3 min., followed by 40 cycles of 15 s at 95 ℃,15 s at 34 ℃, and 15 s at 72 ℃, with a final extension at 72 °C for 8 min. The RAPD products along with a 100 bp DNA Ladder were electrophoresed in 1.5% agarose gel in 1x TAE buffer at 85 V for 45 min. The gels were stained with ethidium bromide (0.5 µg/mL) added to the cooled agarose solution before pouring the gel and visualized under a UV transilluminator after electrophoresis. All RAPD experiments were also performed in triplicate for three populations of each species.

**Table 3:** RAPD primers and analysis of their corresponding banding profiles obtained for the studied *Artemisia* species

Primer Code	Sequence (5’→3’)	Total band number	monomorphic bands	Polymorphic bands	% polymorphism	PIC	EMR	MI	RP
**OPG-02**	5’-GGCACTGAGG-3’	10	0	10	100	0.4125	10	4.125	6.5
**OPG-03**	5’-GAGCCCTCCA-3’	10	0	10	100	0.4	10	4	6
**OPG-04**	5’-AGCGTGTCTG-3’	9	0	9	100	0.3889	9	3.5	5
**OPG-05**	5’-CTGAGACGGA-3’	7	0	7	100	0.4107	7	2.8749	4.5
**OPG-06**	5’-GTGCCTAACC-3’	9	0	9	100	0.3889	9	3.5	5
**OPG-07**	5’-GAACCTGCGG-3’	2	0	2	100	0.5	2	1	2
**OPG-08**	5‘-TCACGTCCAC-3‘	5	0	5	100	0.425	5	2.125	3.5
**OPG-09**	5‘-CTGACGTCAC-3‘	10	0	10	100	0.3625	10	3.625	6.5
**OPA-10**	5’-GTGATCGCAG-3’	15	0	15	100	0.375	15	5.625	7.5
**OPA-11**	5’-CAATCGCCGT-3’	12	0	12	100	0.4062	12	4.8744	7.5
**OPA-09**	5’-GGGTAACGCC-3’	7	0	7	100	0.4107	7	2.8749	5.5
**OPD-15**	5’-CATCCGTGCT-3’	7	0	7	100	0.4107	7	2.8749	5.5
**OPB-19**	5’-ACCCCCGAAG-3’	7	0	7	100	0.4286	7	3	6
**OPAT-19**	5’-ACCAAGGCAC-3	7	0	7	100	0.375	7	2.625	3.5
**OPK-07**	5’-AGCGAGCAAG-3’	7	0	7	100	0.375	7	2.625	3.5
**OPS-12**	5’-CTGGGTGAGT-3’	5	0	5	100	0.45	5	2.25	4
**OPB-13**	5’-TTCCCCCGCT-3’	5	0	5	100	0.4	5	2	4
**OPA-04**	5’-AATCGGGCTG-3’	9	1	8	88.88	0.375	7.11	2.67	6.5
**OPB-17**	5’-AGGGAACGAG-3’	8	0	8	100	0.4062	8	3.2496	5
**OPB-07**	5-GGTGACGCAG-3	6	0	6	100	0.3958	6	2.3748	3.5
**OPA-16**	5’-AGCCAGCGAA-3’	10	0	10	100	0.425	10	4.25	9
**OPB-03**	5’-CATCCCCCTG-3’	6	0	6	100	0.4583	6	2.7498	5
**OPB-20**	5’-GGACCCTTAC-3’	10	0	10	100	0.4	10	4	6
**OPAH-17**	5’-CAGTGGGGAG-3’	6	0	6	100	0.4583	6	2.7498	5
**OPA-02**	5-TGCCGAGCTG-3	8	0	8	100	0.4375	8	3.5	6
**OPA-14**	5’-TCTGTGCTGG-3’	3	0	3	100	0.375	3	1.125	1.5
**OPB-18**	5’-CCACAGCAGT-3’	12	0	12	100	0.3854	12	4.6248	6.5

### SCAR markers development

Distinctive and reproducible RAPD bands for *A. annua*,* A. herba-alba*,* A. monosperma and A. judaica* generated by primers OPG-02, OPG-04, OPA-09 and OPD-15 RAPD primers, respectively, were used for developing SCAR markers. The distinguishable bands (eco-type specific characteristic RAPD markers) were excised from 1.8% agarose gel. Bands were purified using the GeneJET Gel Purification Kit (Thermo Scientific, USA) and cloned into the pJET 1.2/blunt vector using the CloneJET PCR Cloning Kit (Thermo Scientific, USA). Recombinant plasmids were transformed into *Escherichia coli* DH5-α competent cells via heat shock and selected on LB medium supplemented with ampicillin. Successful transformations were confirmed by colony PCR using primers for the pJET 1.2/blunt vector to guarantee correct insertion of the targeted RAPD fragments in the cloned vectors. Plasmids were purified from positive colonies using the GeneJET Plasmid kit (Thermo Scientific, USA).

## Data analysis

### DNA barcoding

Obtained chromatograms of the purified PCR products were trimmed using Chromatogram Explorer Lite 3.2. The consensus sequences were assembled in BioEdit Sequence Alignment Editor Version 7.2.5 using both forward and reverse primers. These assembled sequences were aligned against National Center for Biotechnology Information (NCBI) database using Basic Local Alignment Search Tool (BLAST) tool (https://blast.ncbi.nlm.nih.gov/Blast.cgi). The sequences of both DNA barcodes were deposited in the NCBI GenBank^®^ under accession numbers listed in Table S1. MUSCLE algorithm of Molecular Evolutionary Genetic Analysis (MEGA 11) software was used for the alignment of the constructed sequences for both loci, in addition, phylogenetic trees based on the maximum likelihood method were constructed. The aligned sequences were analysed for conserved/variable/parsimony informative/singleton sites, transition/transversion pairs and indels, to evaluate the discrimination power of each locus.

### ISSR fingerprinting and RAPD profiling

ISSR and RAPD bands were manually scored as present (1) or absent (0) to assess genetic similarity among samples. Binary data matrices were used to estimate primer banding characteristics such as the total number of bands classifying them into monomorphic & polymorphic ones and their percentages. In addition, the primers were assessed in terms of performance/efficiency parameters; PIC (Polymorphic Information Content), EMR (Effective Multiplex Ratio), MI (Marker index) and RP (Resolution Power). These parameters establish the polymorphism level and the differentiation power of primers [20]. Similarity among the studied species was calculated in terms of Jaccard similarity coefficient using PAleontological STtatistics (PAST 4.03) software. Cluster analysis based on unweighted pair-group method with arithmetic mean (UPGMA), employing Jaccard similarity coefficients, was accomplished using PAST 4.03 software and genetic diversity expressed among the studied plants was addressed according to the obtained dendrograms. POPGENE version 1.31 was utilized for genetic diversity estimation expressed in terms of Na (observed number of alleles), Ne (effective number of alleles), H (Nei’s gene diversity) and I (Shannon’s information index).

### SCAR markers analysis

The recombinant plasmids were sequenced bidirectionally using Sanger sequencing with SeqStudio Flex ™ Series Genetic Analyzer (Thermo Scientific, USA) at Colors Medical Lab., Cairo, Egypt, using the forward and reverse sequencing primers provided with CloneJET PCR Cloning Kit. SCAR-specific primers were designed in Clone Manager 9 Professional Edition based on authentic amplicon sequences, excluding the original RAPD primer regions, to enhance specificity and generate a single distinct amplification product (Table 4).

**Table 4:** Sequences of SCAR marker specific primers

Species	Primer	Sequence (5’−3’)	Amplicon length (bp)	Annealing temperature
*A. annua*	A_F	GGAACACGAAGAGCATGAAC	223 bp	53.5 ℃
	A_R	CCATTACGCCACCCACTAC		
*A. herba-alba*	H_F	GTCTGTATGCGCGACTTCC	440 bp	56 ℃
	H_R	ATTGGCCCGTGTAAGCTGTG		
*A. monosperma*	M_F	CGCCACAGAAATTCGTCAG	361 bp	53.4℃
	M_R	AAGCGCAAACACAACGAC		
*A. judaica*	J_F	CGTGCTGCATAAGAACATAG	303 bp	51.8 ℃
	J_R	GATCTCTATGGGCGTGTG		

### SCAR markers validation

The newly designed SCAR primers were tested by PCR to verify genotype specificity and discrimination ability. PCR reactions using the designed specific primer pairs against the studied *Artemisia* species (Table 4), were performed under the ideal cycling parameters, to verify the specificity and assess the applicability of the established SCAR markers for their putative discrimination. Each SCAR primer pair was evaluated against its target species and the other *Artemisia* species to confirm specificity and applicability.

## Results and discussion

### DNA barcoding

DNA barcoding is regarded as a powerful, emerging tool for species identification and authentication. It is a rapid, robust, specific, sensitive, and eco-friendly technique that utilizes a short DNA sequence from a standard region of the genome. Short genomic regions (< 1000 bp), known as “barcodes,” are used to distinguish closely related species where the resulting DNA barcodes are blasted against different reference databases for sample authentication and verification [21–23]. DNA barcoding may be single-locus or multi-locus (tiered approach), targeting both plastid and nuclear loci to improve species identification and discrimination. Combining two or more loci, usually plastid and nuclear, is often essential to achieve higher levels of species differentiation and to support phylogenetic and evolutionary studies [24].

Liu et al. utilized seven DNA barcodes (ITS, ITS2, psbA-trnH, rbcL, matK, rpoB, and rpoC1) to identify the closely related *Artemisia* species; *A. annua*,* A. absinthiu*,* A. rupestris*,* A. tonurnefortiana*,* A. austriaca*,* A. dracunculus*,* A. vulgaris*,* A. gmelinii*,* A. anethoides*,* A. pubescens*,* A. macrocephala*,* A. scoparia*,* A. sieversiana*,* and A. pontica* in Xinjiang, China [25]. Also, *A. maritima and A. absinthium* were subjected to chloroplast genome sequencing due to unresolved taxonomic ambiguity at the species level. Both species displayed high similarity in genome size, gene synteny, GC content, transition to transversion ratio below 1; twenty polymorphic regions were identified to help resolve their taxonomic distinctions [26]. Additionally, Wang et al. reported that direct PCR sequencing of the ITS2 region provided reliable results by identifying misreads and deletion sites within the ITS region, supporting ITS2 as a preferable barcode for *Artemisia* species [27]. The psbA-trnH intergenic spacer is also among the most variable regions in the chloroplast genome of angiosperms. Its high discriminatory power and extra variation makes it a recommended complement to ITS for plant DNA barcoding [28].

**Fig. 1:**
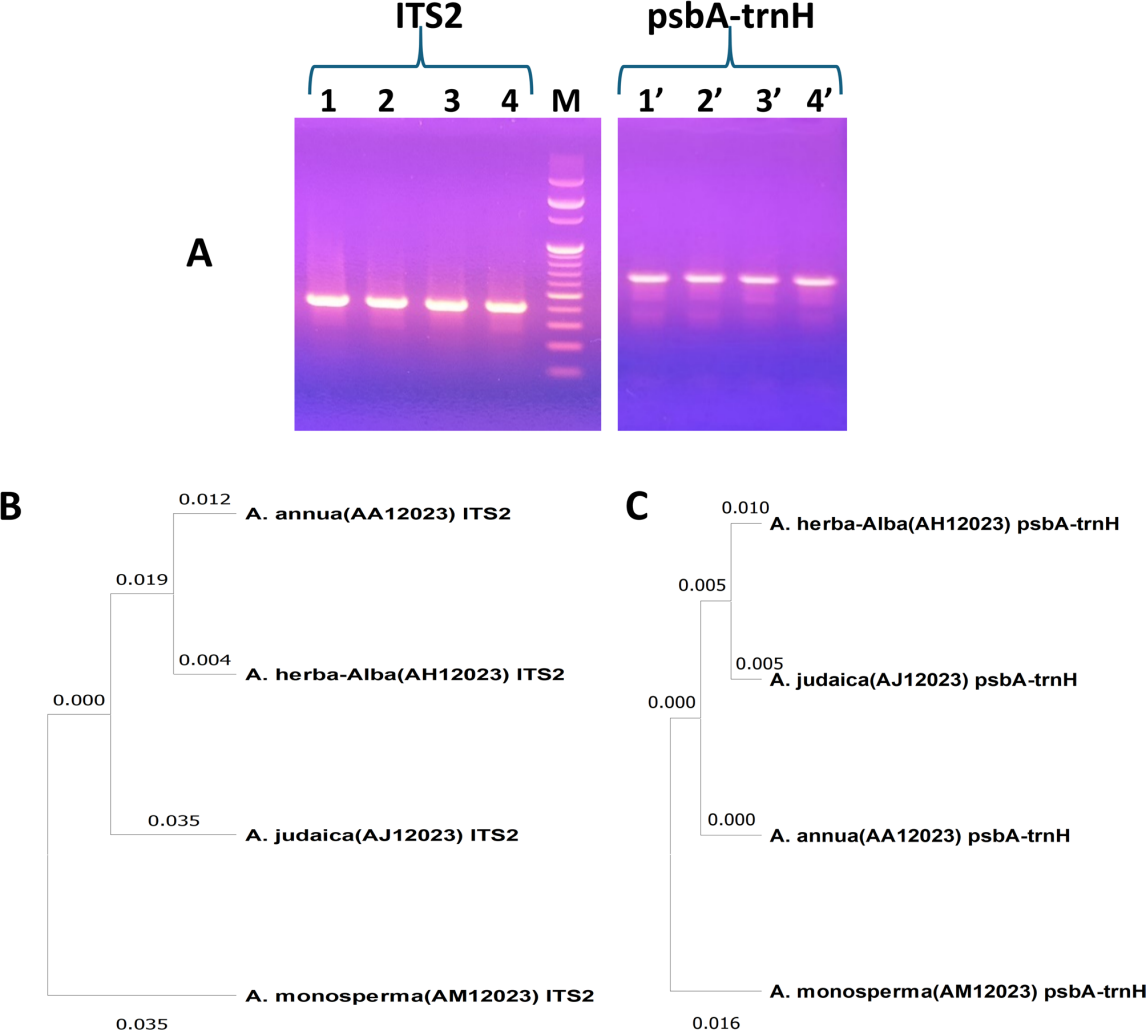
**(A) **The resultant PCR products of* A. annua* (1 and 1'), *A. herba-alba* (2 and 2'), *A. monosperma* (3 and 3') and *A. judaica* (4 and 4') for the targeted DNA barcodes; ITS2 and psbA-trnH, respectively, loaded against 100 bp DNA ladder (lane M), combined with the phylogenetic trees of the studied *Artemisia* species constructed based on **(B)** ITS2 and **(C)** psbA-trnH sequences using the Maximum Likelihood method with Kimura 2-parameter model employing discrete Gamma distribution for modelling evolutionary rate differences among sites and Tamura 3-parameter model, respectively. The tree was obtained automatically by applying Neighbor-Join and BioNJ algorithms comprising 4 nucleotide sequences with total of 444 and 547 positions, respectively, in the final dataset.

Based on these reports, the nuclear ITS2 and plastid psbA-trnH spacers were targeted in this study as potential DNA barcodes for authenticating the Egyptian *Artemisi*a species (*A. herba-alba*, *A. monosperma* and *A. judaica*) in addition to cultivated *A. annua*, yielding sequences of approximately 400 and 500 bp, respectively (Fig. 1A).

For each barcoding locus, the assembled sequences were compared against the NCBI database using BLAST tool for species verification. The ITS2 BLAST results accurately identified *A. annua*,* A. herba-alba*,* A. monosperma and A. judaica* with percentage identities of 99.5%, 99.22%, 99.24% and 99.68%, respectively. The alignments showed appropriate query coverage and percentage identity exceeding 99%, confirming successful and accurate species identification based on their ITS2 sequences. For the plastid psbA-trnH intergenic spacer, BLAST results correctly identified *A. annua* and *A. herba-alba* with percentage identities of 99.2% and 99.56%, respectively, and query coverage exceeding 90%. However, psbA-trnH sequences for *A. monosperma* and *A. judaica* did not match reference sequences at the species level but were assigned to the genus *Artemisia*.

Alignment of ITS2 and psbA-trnH sequences showed that ITS2 contained more variable sites than psbA-trnH (Figs. S1 and S2), with a predominance of singleton sites over parsimony-informative sites, indicating higher species-level discrimination. In contrast, psbA-trnH showed more conserved sites, fewer variable positions, and more indels (Table S2). Further analysis revealed that transversions were more frequent in psbA-trnH leading to transition to transversion ratio (R = si/sv) value less than 1. Whereas transitions exceeded transversions in ITS2 sequences with ratio (R = si/sv) of 4 (Table S2).

Phylogenetic Neighbour-Joining (NJ) trees were constructed and evaluated to assess genetic distances and the discriminatory power of each barcode. The ITS2-based NJ tree (Fig. 1B) divided *Artemisia* species into two clades; a clade for *A. monosperma* and another clade where *A. judaica* is in a discrete subclade while the other subclade grouped *A. annua* at a genetic distance of 0.035 from *A. herba-alba.* Also, *A. annua* was separated from both *A. judaica* and *A. monosperma* by a genetic distance of 0.066.

The psbA-trnH based NJ tree (Fig. 1C) aligned more closely with morphological traits, grouping *A. herba-alba* and *A. judaica* together. The tree displayed two clades; one for *A. monosperma* and another comprised *A. annua* in a separate subclade, separated from *A. herba-alba* and *A. judaica* by a genetic distance of 0.015 and 0.01, respectively. *A. herba-alba* and *A. judaica* were grouped together in the other subclade separated by a distance of 0.02. This is consistent with findings by the CBOL Plant Working Group, which reported that psbA-trnH has high discriminatory power and is recommended as the most favoured complementary locus alongside matk locus [24].

In both trees, *A. monosperma* formed a distinct clade with the other species grouped differently in the other clade. According to psbA-trnH, *A. herba-alba* and *A. judaica* were separated by a genetic distance of 0.02, compared to 0.058 based on ITS2 sequences, suggesting that ITS2 provided clearer separation of these morphologically and chemically similar species. In addition, *A. annua* was also better resolved from the other species based on ITS2 sequences. Therefore, ITS2 was recommended over psbA-trnH as the preferred discriminatory barcode for efficient authentication and differentiation of these *Artemisia* species. These results support ITS2’s reputation as one of the most widely used phylogenetic markers for eukaryotes and a universal DNA barcode for plants, and as an alternative locus to the mitochondrial cytochrome oxidase subunit 1 (CO1) for animal species identification [29].

### ISSR fingerprinting

Inter simple sequence repeat (ISSR) fingerprinting is a PCR-based method using microsatellite primers to produce highly specific multi-locus markers. Microsatellites are highly polymorphic, abundant, easily amplified by standard PCR, and evenly distributed throughout the euchromatic genome regions. These characteristics make microsatellites one of the most valuable and comprehensive genetic markers for genome mapping, paternity testing, population genetics, and studies of genetic diversity [30, 31]. ISSR amplifies DNA regions present in between two identical simple-sequence repeats (SSRs). Their widespread distribution throughout the genome allows detection of variations due to insertions or deletions, providing strong discriminatory power [32–34]. High reproducibility of this technique is ensured by longer primer lengths and higher annealing temperatures (45–60 °C) [35].

Eighty individuals from eight *A. herba-alba* populations, growing wild in numerous geographic zones in Tunisia, were analysed for intraspecific variation in essential oil content, genetic diversity, and population structure using ISSR and RAPD markers. The study revealed high within-population genetic diversity and strong correlations between chemical and molecular markers [36]. In 2021, Al-Ajmi et al. investigated the genetic diversity of seven *Artemisia* species (*A. ludoviciana* Nutt, *A. princeps* pamp, *A. scoparia* walst and kitam, *A. annua* L., *A. monosperma* Del., *A. frigida* wild and *A. herba-alba* Asso) using ISSR fingerprinting. The study demonstrated the effectiveness of ISSR primers in detecting polymorphism [37]. Gaafar et al. also reported polymorphism and Nei’s genetic diversity for seven *A. judaica* populations collected from South Sinai, Egypt, using ISSR [38].

**Fig. 2:**
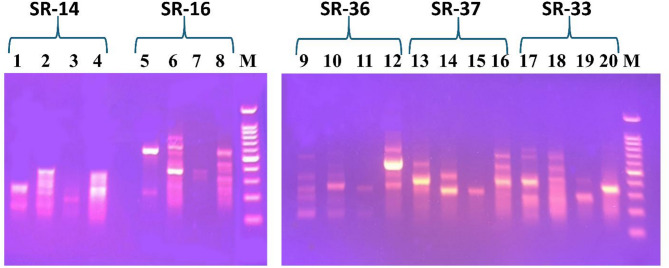
ISSR profiles of the studied *Artemisia* species using 5 ISSR primers; SR-14, SR-16, SR-36, SR-37 and SR-33, loaded against 100 bp DNA ladder (lane M). *A. annua* was loaded in (lanes 1, 5, 9, 13 and 17), *A. herba-alba* was loaded in (lanes 2, 6, 10, 14 and 18), *A. monosperma* was represented by (lanes 3, 7, 11, 15 and 19) *and A. judaica* by (lanes 4, 8, 12, 16 and 20). Original uncropped gels are presented in Supplementary Figure S3

In this study, five selected ISSR primers generated reproducible polymorphic profiles (Fig. 2), producing a total number of 41 polymorphic bands with 100% polymorphism. SR-16 and SR-36 generated the most fragments (10 bands), while SR-37 generated the fewest (6 bands), averaging 8.2 bands per primer (Table 2). The average polymorphism information content (PIC) value was 0.42, with SR-14 showing the highest PIC (0.4464) and SR-16 the lowest (0.4). Primers SR-16 and SR-36 showed the highest effective multiplex ratio (EMR) value reflecting the highest differentiation power, and primer SR-36 displayed the highest marker index (MI) value of 4.375. Resolving power (RP) ranged from 4 (SR-37) to 8.5 (SR-36) (Table 2).

Binary data from banding profiles were analysed using PAST 4.03 to estimate genetic similarity via Jaccard’s similarity coefficients (Table S3), visualized as dendrograms (Table S4). The estimated Jaccard’s similarity index ranged from 0.0322 to 0.2963. *A. herba-alba and A. judaica* depicted low genetic diversity and a close evolutionary relationship.

Dendrograms confirmed that *A. judaica* could be successfully distinguished from its morphologically and chemically resembling species; *A. herba-alba* [8, 11], using primers SR-33 and SR-37. Primer SR-33 placed *A. judaica* in a distinct UPGMA clade without any genetic similarity to the other studied *Artemisia* species, while primer SR-37 grouped *A. judaica* with *A. annua* with a genetic similarity of 25%. Similarly, primer SR-16 offered the same advantage of complete distinguishing of *A. annua* and *A. monosperma*, each in their own clade. On the other hand, primer SR-14 was not recommended as it displayed the highest similarity among *A. judaica* and *A. herba-alba* (60%) despite clear differentiation of *A. monosperma* (Table S3). SR-36 and SR-16 clustered *A. herba-alba* and *A. judaica* together, matching their morphological similarity (Table S4). Thus, primer SR-33 was recommended as primer of choice for *A. judaica* discrimination. *A. monosperma* and *A. annua* could be completely distinguished using ISSR primer SR-16. ISSR primer SR-36 was recommended for general *Artemisia* species differentiation owing to its high PIC, EMR, MI and RP values. Primer SR-16 closely followed SR-36, exhibiting high performance/efficiency parameters. However, both primers showed high genetic similarity among *A. herba-alba* and its closely related species; *A. judaica.* Therefore, a complementary genetic profiling technique was recommended for better differentiation of these two related species.

The binary data generated from different populations of the studied *Artemisia* species with the five selected ISSR primers were combined to assess genetic similarity among them in terms of Jaccard’s similarity coefficient. The estimated Jaccard’s similarity index ranged from 0.0322 to 0.2963. The similarity index between *A. herba-alba and A. judaica* depicted low genetic diversity and closer evolutionary relationship between them based on their ISSR banding profiles (Table S5). Cluster analysis using UPGMA was conducted with the estimated similarity index and displayed as dendrogram in Fig. S4. The UPGMA tree based on Jaccard’s similarity coefficient (Fig. S4) separated *A. annua* and *A. monosperma* into one main clade and *A. herba-alba* and *A. judaica* into another, showing partial differentiation of these related species.

The level of genetic diversity revealed by ISSR markers varied among the studied plant species. Based on ISSR analysis, the observed number of alleles (Na) ranged from 1.2439 to 1.3415. The effective number of alleles (Ne) varied between 1.1951 and 1.2732. The percentage of polymorphic loci (PPL) ranged from 24.39 to 34.15%. Shannon’s index (I) for the four species ranged from 0.1552 to 0.2173, while Nei’s gene diversity (H) varied between 0.1084 and 0.1518 (Table S6).

These findings confirm that the ISSR technique was effective for revealing genetic relationships among *Artemisia* species and allowed partial discrimination between the morphologically and chemically similar *A. herba-alba* and *A. judaica*. Although some ISSR primers clearly distinguished certain species, the clustering patterns indicated that additional complementary techniques are necessary for complete and reliable differentiation, particularly for species with high morphological and chemical similarity.

### RAPD profiling

Randomly amplified polymorphic DNA (RAPD-PCR) is a molecular technique widely applied for the quality control and authentication of botanical materials. It is popular for its affordability and effectiveness in studying intraspecific genetic variation. RAPD uses short, random primers to bind non-specific DNA regions and amplify them [39, 40]. RAPD primers are arbitrarily designed so the technique samples the genome randomly, generating multi-locus banding patterns that can be easily scored and converted into single-locus markers. This method requires minimal plant material and does not rely on prior sequence information. RAPD is rapid and cost-effective, producing large numbers of bands that are useful for detecting genetic polymorphisms [32, 33, 41].

RAPD has been increasingly used for the verification and authentication of medicinal plants including different *Artemisia* species [34]. Elmeer and Elkhgkheg investigated *A. herba-alba* populations from various altitudes in Libya’s Green Mountain using sequence related amplified polymorphism (SRAP) and RAPD, revealing that genetic relationships were not strongly correlated with their geographical origin [42]. Similarly, the genetic diversity of *Artemisia* populations in central and northern Saudi Arabia was assessed and differentiated *A. herba-alba* from populations of *A. judaica* and *A. monosperma* using morphological traits and RAPD polymorphism. Both methods revealed that *A. herba-alba* was more polymorphic than the other two species [43].

In this study, RAPD profiling was performed, complementary to ISSR fingerprinting, to enhance species discrimination. Reproducible PCR amplification patterns (banding profiles) were generated (Fig. S5 and S6) using the 27 RAPD primers listed in Table 3, with amplification repeated in triplicates to confirm reproducibility. A total of 212 bands were produced, of which 211 were found to be polymorphic (99.53%) and one was monomorphic (0.47%), with amplicon sizes ranging from 100 to 1000 bp. Primer OPA-10 generated the highest number of bands (15), while primer OPG-07 produced the fewest (4 bands) and primer OPA-04 generated only one monomorphic band. On average, the number of bands was 7.85 and polymorphic bands was 7.81 per primer. The percentage of polymorphic bands ranged from 88.88% (OPA-04) to 100% for the other 26 primers. The average PIC was 0.4087, with the highest PIC value (0.5) for primer OPG-07 and the lowest (0.3625) for primer OPG-09. Primer OPA-10 showed the highest EMR and MI values. The resolution power of the RAPD primers ranged from 1.5 (OPA-14) to 9 (OPA-16) (Table 3).

Jaccard’s similarity coefficient was utilized to estimate genetic similarity among the studied *Artemisia* species, calculated in Table S7. The resulting similarity matrices were then utilized to generate dendrograms illustrating the clustering patterns among the studied species (Table S8). Dendrograms generated using primers OPG-02, OPG-03, OPG-04, OPG-05, OPG-06, OPG-09, OPA-09, OPB-19, OPS-12, OPA-04, OPB-07, OPA-16 and OPAH-17 consistently grouped *A. herba-alba* and *A. judaica* within the same clade or subclade (Table S8). Banding profiles for *A. herba-alba* and *A. judaica* obtained using primers OPG-07 and OPB-20 failed to distinguish these two species. However, several primers resulted in banding profiles with similarity matrix capable of resolving *A. judaica* from *A. herba-alba* successfully. *A. herba-alba* could be differentiated apart from the other *Artemisia* species using primers OPA-10, OPA-11, OPK-07 and OPB-13. Additionally, *A. judaica* could be successfully and totally segregated using primers OPA-10, OPB-18, OPB-03, OPK-07 and OPD-15 (Table S8). Dendrograms created from primers OPG-03, OPG-04, OPG-06, OPG-09, OPA-10, OPK-07, OPB-07 and OPB-20 banding profiles effectively separated *A. annua* into its own clade. Primers OPG-02, OPG-03, OPG-04, OPG-06, OPA-10, OPA-11, OPK-07, OPB-07, OPB-18 and OPB-20 successfully distinguished *A. monosperma* from the other species, whereas OPS-12 failed to separate *A. annua* from *A. monosperma*, and OPD-15 failed to distinguish *A. monosperma* from *A. herba-alba* (Table S8). Overall, RAPD primers OPA-10 and OPK-07 were recommended for reliably differentiating the studied *Artemisia* species, especially *A. herba-alba* from its morphologically and chemically resembling species; *A. judaica*, without any genetic similarity. On the other hand, primers OPG-07, OPB-20, OPS-12 and OPD-15 were not recommended owing to their failure in differentiating the studied species.

Combined RAPD data from the 27 primers were further analysed for multivariate genetic similarity. The calculated Jaccard’s similarity coefficients (Table S9) were used to construct UPGMA based tree/dendrogram (Fig. S7) reflecting relationships among the species. The estimated Jaccard’s Similarity index between the *Artemisia* species ranged from 0.0582 to 0.2098. The highest similarity index was observed between *A. herba-alba* and *A. judaica* (20.98%), indicating low genetic diversity and a close evolutionary link (Table S9). The UPGMA dendrogram (Fig. S7) divided the *Artemisia* species into two main clades; one for *A. annua* and the other comprised two subclades for *A. monosperma*, and for *A. herba-alba* and *A. judaica*, which were grouped together but clearly distinguished from each other.

Based on RAPD analysis, the observed number of alleles (Na) varied from 1.3019 to 1.3396. The effective number of alleles (Ne) ranged between 1.2415 and 1.2717. The percentage of polymorphic loci (PPL) ranged from 30.19 to 33.96%. Shannon’s index (I) of the studied *Artemisia* species ranged from 0.1922 to 0.2162, while Nei’s gene diversity (H) ranged between 0.1342 and 0.1509. The number of polymorphic loci (PL) spanned from 64 to 72 (Table S10).

### SCAR markers

Sequence-characterized amplified region (SCAR) markers derived from various profiling techniques, are quick and reliable tools for authenticating valuable commercial plants and detecting adulteration. Thus, SCAR markers play a crucial role in quality control protocols by ensuring purity, quality and efficacy while minimizing the need for extensive phytochemical and analytical testing of samples [44, 45]. Combining RAPD and SCAR markers offers a simple and efficient strategy for the genetic characterization of plant species. SCAR markers developed from RAPD overcome the reproducibility challenges associated with RAPD, which uses short primers with low annealing temperatures that can cause inconsistent polymorphism patterns. In other words, by converting RAPD markers into SCAR markers, they become more robust, specific, and reproducible [32, 33]. Thus, SCAR markers are now widely recognized as one of the best marker systems for crop authentication and genotype identification [46–49].

Technically, a unique RAPD fragment is isolated, cloned, and sequenced to develop a SCAR marker. A specific primer pair is then designed based on the nucleotide sequence of the RAPD fragment. Once validated, SCAR markers enable rapid screening of large sample sets with high accuracy and reproducibility. SCAR analysis reduces the number of reactions needed for fingerprinting to a single, highly specific assay that produces clear bands suitable for automated analysis, enhancing species identification and purity evaluation [49].

**Fig. 3:**
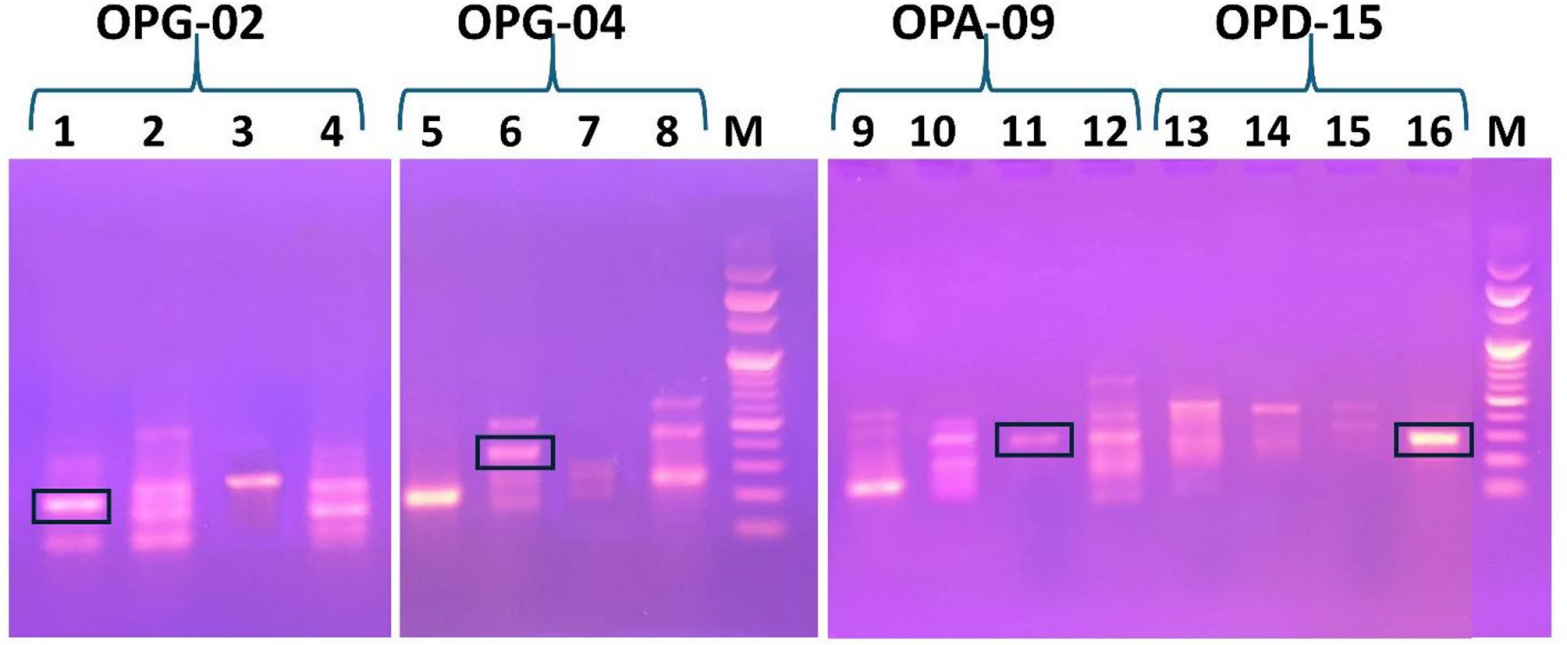
RAPD banding profiles of the studied *Artemisia* species using OPG-02, OPG-04, OPA-09 and OPD-15 for targeting SCAR markers (indicated by boxes) of *A. annua* (lane 1), *A. herba-alba* (lane 6), *A. monosperma* (lane 11) *and A. judaica* (lane 16), along with 100 bp DNA ladder (lane M). Original uncropped gels are presented in Supplementary Figure S6

**Fig. 4:**
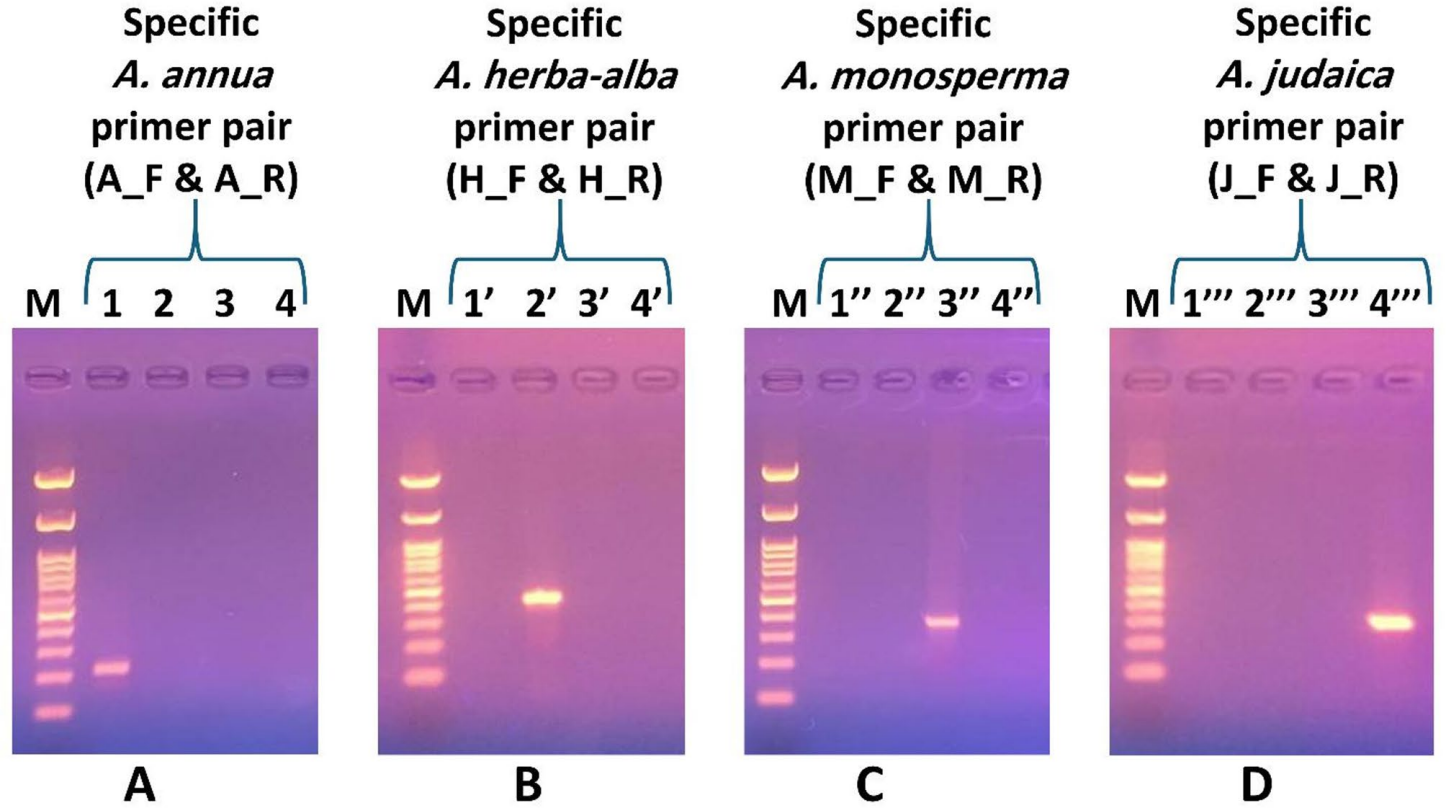
The resultant RAPD based SCAR markers for samples of *A. annua* (Lanes 1, 1’, 1’’ and 1’’’), *A. herba-alba* (lanes 2, 2’, 2’’ and 2’’’), *A. monosperma* (lanes 3, 3’, 3’’and 3’’’) and *A. judaica* (lanes 4, 4’, 4’’ and 4’’’) using the designed specific primer pairs, loaded against 100 bp DNA ladder (lane M). Original uncropped gels are presented in Supplementary Figure S10

In this study, reproducible RAPD profiles for *A. annua*,* A. herba-alba*,* A. monosperma* and *A. judaica* generated with OPG-02, OPG-04, OPA-09 and OPD-15 RAPD primers, respectively, were targeted for SCAR marker development (Fig. 3). Specific RAPD fragments were tracked, purified, cloned, and sequenced. Based on these sequences, unique primer pairs were designed. The sequence data for the resulting ecotype-specific RAPD-derived SCAR markers were deposited in NCBI GenBank^®^ under the accession numbers listed in Table S11. The sequence of the designed primer pairs, their corresponding amplicons’ sizes and optimum annealing temperatures are provided in Table 4. The developed SCAR markers were assessed and validated using multiple samples of each species, where all the examined samples for the same species showed a single, species-specific SCAR upon using its corresponding designed primer pair confirming its reproducibility (Fig. S8 and S9). For each designed primer pair, SCAR marker was produced exclusively for its corresponding species and no band was generated for the other species as shown in Fig. 4. To clarify, the SCAR primers for *A. annua* (A_F and A_R) amplified a 223 bp product exclusively in *A. annu*a samples, with no bands for the other *Artemisia* species (Fig. 4A). Similar patterns of results were observed when the other designed SCAR specific primer pairs were tested for detection of their corresponding *Artemisia* species (Fig. 4B, C and D). This demonstrates the high selectivity and specificity of the developed SCAR markers for their corresponding species and confirms their potential for reliable species authentication and discrimination.

## Conclusion

A tiered DNA barcoding approach was implemented, targeting both plastid and nuclear spacers - psbA-trnH and ITS2, respectively- aiming at precise authentication of the studied *Artemisia* species. ITS2 was recommended over psbA-trnH as the preferred discriminatory barcode, as it allowed for more specific and efficient authentication and differentiation of the species. This was confirmed by constructing a phylogenetic tree, which showed clearer clustering and greater genetic distances, facilitating species differentiation. ISSR fingerprinting and RAPD profiling were also employed to discriminate the studied *Artemisia* species, based on their reproducible banding profiles generated using 5 ISSR primers and 27 RAPD primers, respectively. This approach helped unravel the genetic similarity and diversity within the studied *Artemisia* species. Among the RAPD primers, OPA-10 and OPK-07 were recommended for their superior discriminatory power. The developed species-specific RAPD-based SCAR markers were successfully applied for authentication and discrimination of the examined *Artemisia* species, with validated putative nature confirming selectivity and specificity. It is worth mentioning that this is the first report of eco-specific SCAR markers for the Egyptian *Artemisia* species, which can be used for routine authentication in quality control protocols, enabling the simultaneous analysis of large numbers of samples.

## Supplementary Information

Below is the link to the electronic supplementary material.

Supplementary Material 1

Supplementary Material 2

Supplementary Material 3

Supplementary Material 4

## Data Availability

The data supporting this article have been included in the manuscript and supplementary information. Additional data will be made available upon reasonable request to the corresponding author.

## Abbreviations

BLAST: Basic local alignment search tool
CBOL: Consortium for the barcodes of life
EMR: Effective multiplex ratio
H: Nei’s gene diversity index
I: Shannon’s information index
ISSR: Inter-simple sequence repeat
ITS2: Internal transcribed spacer 2
MEGA: Molecular evolutionary genetic analysis
MI: Marker Index
Na: Observed number of alleles
NCBI: National center for biotechnology information
Ne: Effective number of alleles
PAST: PAleontological STatistics software
PIC: Polymorphism information content
RAPD: Random amplified polymorphic DNA
RP: Resolution power
SCAR: Sequence characterized amplified regions
PL: Number of polymorphic loci
PPL: Percentage of polymorphic loci
UPGMA: Unweighted pair group method with arithmetic mean
