# Supplementary figures and images for: Unravelling Artemisia species genetic variation via DNA barcoding, ISSR and RAPD with the development of eco-specific SCAR markers
Source: BMC Plant Biol. 2025 Aug 7;25:1034. doi: 10.1186/s12870-025-07058-9 (PMC12329950; doi:10.1186/s12870-025-07058-9)

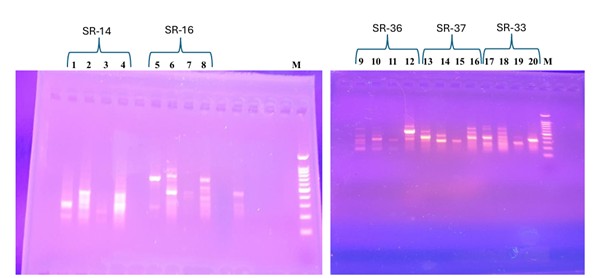

Supplement: Supplementary file 1 — Supplementary Material 1 [file 12870_2025_7058_MOESM1_ESM.jpg]

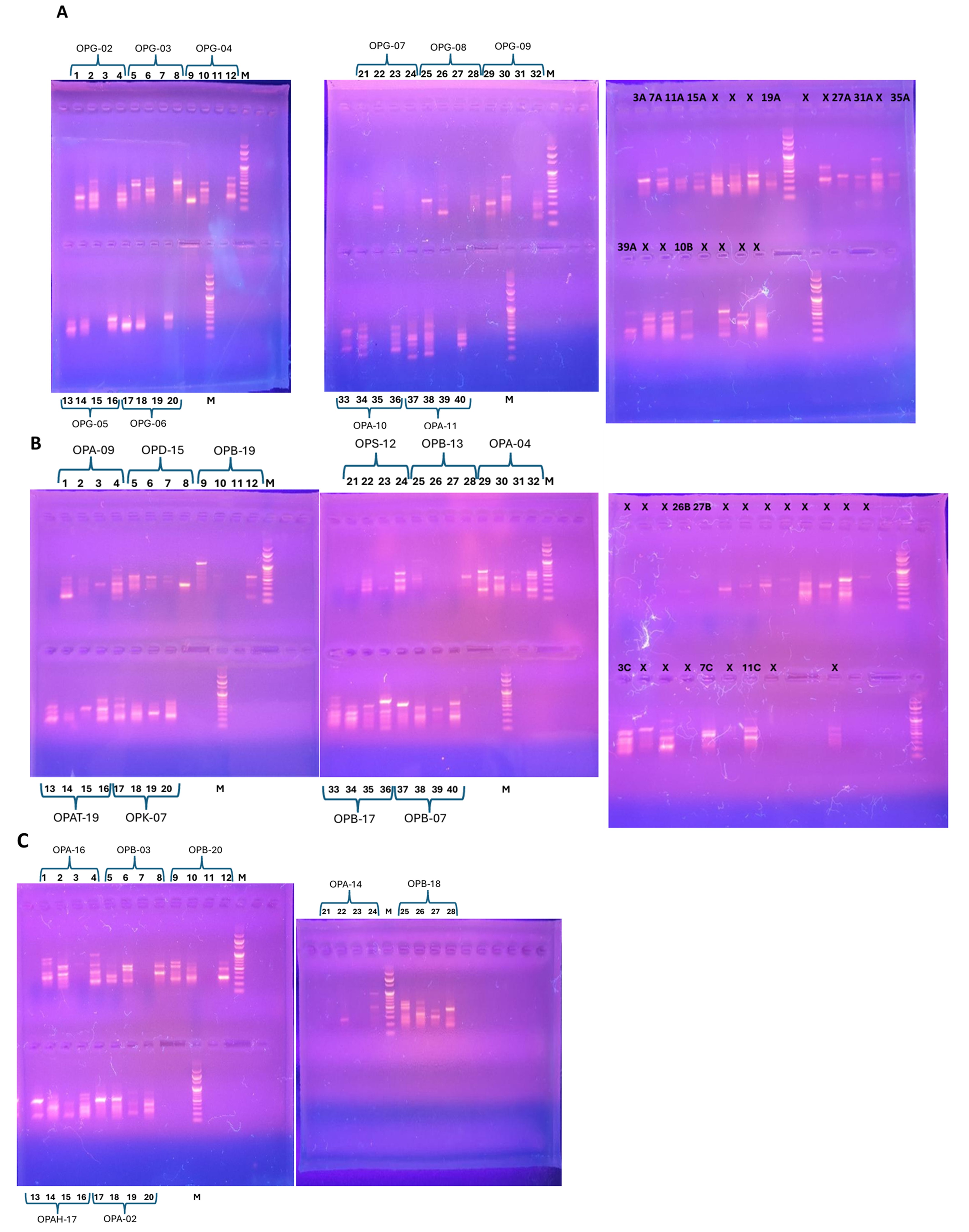

Supplement: Supplementary file 2 — Supplementary Material 2 [file 12870_2025_7058_MOESM2_ESM.jpg]

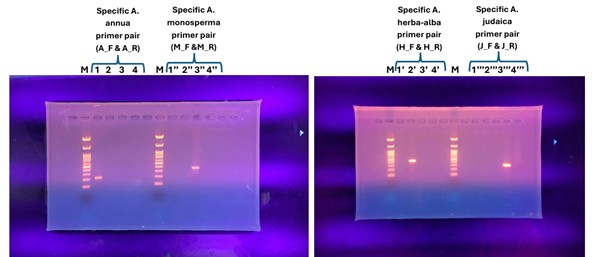

Supplement: Supplementary file 3 — Supplementary Material 3 [file 12870_2025_7058_MOESM3_ESM.jpg]
