# Supplementary material for: Unravelling Artemisia species genetic variation via DNA barcoding, ISSR and RAPD with the development of eco-specific SCAR markers
Source: BMC Plant Biol. 2025 Aug 7;25:1034. doi: 10.1186/s12870-025-07058-9 (PMC12329950; doi:10.1186/s12870-025-07058-9)
Supplement: Supplementary file 4 — Supplementary Material 4 [file 12870_2025_7058_MOESM4_ESM.pdf]

Supplementary data

Unravelling *Artemisia* species genetic variation via DNA barcoding, ISSR and RAPD with the development of eco-specific SCAR markers

Yasmin A. Mahgoub[1,†], Hebaalla A. Mahmoud[1,†], Nadia A. El-Sebakhy[1], Ingy I. Abdallah[1,*]

[1] Department of Pharmacognosy, Faculty of Pharmacy, Alexandria University, Egypt.

[†] Y.A.M. and H.A.M contributed equally to this work.

[*] Corresponding author: Dr. Ingy I. Abdallah, Department of Pharmacognosy, Faculty of Pharmacy, Alexandria University, Alexandria 21521, Egypt.

E-mail: ingy.ibrahim@alexu.edu.eg

Table S1 GenBank accession numbers for ITS2 and psbA-trnH spacers of the studied plants

Plant samples	Sample code	ITS2 accession number	psbA-trnH accession number
A. annua	AA12023	OR646583	OR743704
	AA22023	OR646584	OR743705
	AA32023	OR646585	OR743706
A. herba-alba	AH12023	OR646586	OR743707
	AH22023	OR646587	OR743708
	AH32023	OR646588	OR743709
A. monosperma	AM12023	OR646589	OR743710
	AM22023	OR646590	OR743711
	AM32023	OR646591	OR743712
A. judaica	AJ12023	OR646592	OR743713
	AJ22023	OR646593	OR743714
	AJ32023	OR646594	OR743715

Table S2 Sequences comparison of the obtained DNA barcodes of the studied *Artemisia* species.

DNA barcode locus	Conserved sites	Variable sites	Parsimony informative sites	Singleton sites	Transition pairs (si)	Transversion pairs (sv)	transition to transversion ratio (R=si/sv)	Indels
ITS2	379/444	29/444	5/444	20/444	12	3	4	60
psbA-trnH	488/547	16/547	4/547	12/547	4	5	0.8	69

Table S3 Jaccard Similarity Coefficient of ISSR primers.

Primer SR-14

	A. annua	*A. herba-alba*	*A. monosperma*	*A. judaica*
A. annua	1	0.1667	0	0
A. herba-alba	0.1667	1	0	0.6
A monosperma	0	0	1	0
A. judaica	0	0.6	0	1

Primer SR-16

	A. annua	*A. herba-alba*	*A. monosperma*	*A. Judaica*
A. annua	1	0	0	0
A. herba-alba	0	1	0	0.3333
A monosperma	0	0	1	0
A. judaica	0	0.3333	0	1

Primer SR-33

	A. Annua	*A. herba-alba*	*A. monosperma*	*A. judaica*
A. annua	1	0.1428	0.1667	0
A. herba-alba	0.1428	1	0.25	0
A monosperma	0.1667	0.25	1	0
A. judaica	0	0	0	1

Primer SR-36

	A. annua	*A. herba-alba*	*A. monosperma*	*A. Judaica*
A. annua	1	0.25	0.4	0
A. herba-alba	0.25	1	0.1667	0.4285
A monosperma	0.4	0.1667	1	0
A. judaica	0	0.4285	0	1

Primer SR-37

	A. Annua	*A. herba-alba*	*A. monosperma*	*A. Judaica*
A. annua	1	0	0	0.25
A. herba-alba	0	1	0.5	0
A monosperma	0	0.5	1	0
A. judaica	0.25	0	0	1

Table S4 UPGMA trees based on Jaccard's similarity coefficients of ISSR primers for the studied *Artemisia* species

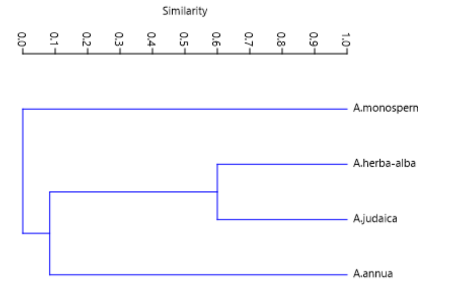

Primer SR-14

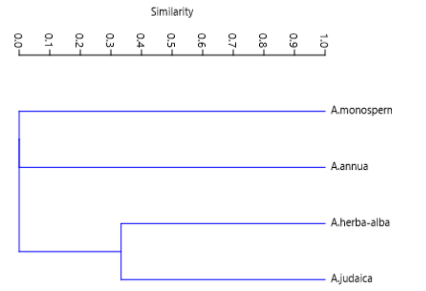

Primer SR-16

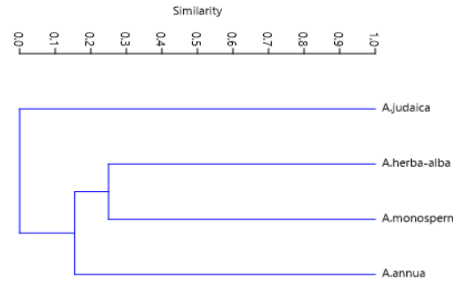

Primer SR-33

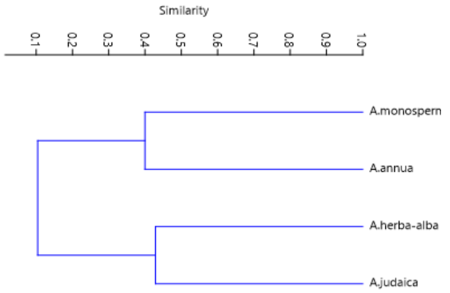

Primer SR-36

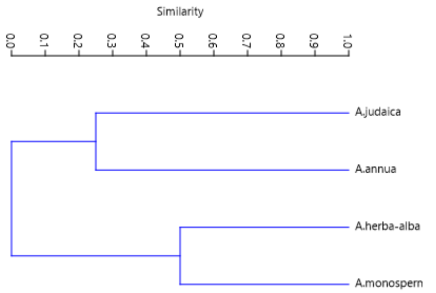

Primer SR-37

Table S5 Collective Jaccard's similarity coefficient of the five selected ISSR primers.

	A. annua (A.A1 2023)	***A. herba-alba*** (A.H1 2023)	***A. monosperma*** (A.M1 2023)	***A. judaica*** (A.J1 2023)
A. annua (A.A1 2023)	1	0.129	0.1428	0.0322
A. herba-alba (A.H1 2023)	0.129	1	0.125	0.2963
A. monosperma (A.M1 2023)	0.1428	0.125	1	0
A. judaica (A.J1 2023)	0.0322	0.2963	0	1

Table S6 Diversity parameters exhibited among different populations of the studied *Artemisia* species based on ISSR profiles.

**Plants**	**Na**	**Ne**	**H**	**I**	**PL**	**PPL**
A. annua	1.2439 (0.4348)	1.1951 (0.3478)	0.1084 (0.0193)	0.1552 (0.0277)	10	24.39%
A. herba-alba	1.2683 (0.4486)	1.2146 (0.3589)	0.1192 (0.0199)	0.1708 (0.0285)	11	26.83%
A. monosperma	1.3415 (0.4801)	1.2732 (0.3841)	0.1518 (0.0213)	0.2173 (0.0306)	14	34.15 %
A. judaica	1.2927 (0.4606)	1.2341 (0.3685)	0.1301 (0.0205)	0.1863 (0.0293)	12	29.27%

Observed number of alleles (Na), Effective number of alleles (Ne), Nei's gene diversity index (H), Shannon's information index(I), Number of polymorphic loci (PL) and Percentage of polymorphic loci (PPL) between populations and between species.

Table S7 Jaccard similarity matrix of the studied *Artemisia* species with different RAPD primers.

Primer OPG-02

	A. annua	*A. herba-alba*	*A. monosperma*	*A. Judaica*
A. annua	1	0.1667	0	0
A. herba-alba	0.1667	1	0	0.2857
A monosperma	0	0	1	0
A. judaica	0	0.2857	0	1

Primer OPG-03

	A. annua	*A. herba-alba*	*A. monosperma*	*A. judaica*
A. annua	1	0	0	0
A. herba-alba	0	1	0	0.5
A monosperma	0	0	1	0
A. judaica	0	0.5	0	1

Primer OPG-04

	A. annua	*A. herba-alba*	*A. monosperma*	*A. Judaica*
A. annua	1	0	0	0
A. herba-alba	0	1	0	0.1667
A monosperma	0	0	1	0
A. judaica	0	0.1667	0	1

Primer OPG-05

	A. annua	*A. herba-alba*	*A. monosperma*	*A. judaica*
A. annua	1	0	0.25	0
A. herba-alba	0	1	0	0.3333
A monosperma	0.25	0	1	0
A. judaica	0	0.3333	0	1

Primer OPG-06

	A. annua	*A. herba-alba*	*A. monosperma*	*A. judaica*
A. annua	1	0	0	0
A. herba-alba	0	1	0	0.2
A monosperma	0	0	1	0
A. judaica	0	0.2	0	1

Primer OPG-07

	A. annua	*A. herba-alba*	*A. monosperma*	*A. judaica*
A. annua	0	0	0	0
A. herba-alba	0	1	0	1
A monosperma	0	0	0	0
A. judaica	0	1	0	1

Primer OPG-08

	A. Annua	*A. herba-alba*	*A. monosperma*	*A. judaica*
A. annua	1	0.3333	0	0
A. herba-alba	0.3333	1	0	0
A monosperma	0	0	1	0.5
A. judaica	0	0	0.5	1

Primer OPG-09

	A. annua	*A. herba-alba*	*A. monosperma*	*A. judaica*
A. annua	1	0	0	0
A. herba-alba	0	1	0.25	0.2857
A monosperma	0	0.25	1	0
A. judaica	0	0.2857	0	1

Primer OPA-10

	A. annua	*A. herba-alba*	*A. monosperma*	*A. judaica*
A. annua	1	0	0	0
A. herba-alba	0	1	0	0
A monosperma	0	0	1	0
A. judaica	0	0	0	1

Primer OPA-11

	A. annua	*A. herba-alba*	*A. monosperma*	*A. judaica*
A. annua	1	0	0	0.6
A. herba-alba	0	1	0	0
A monosperma	0	0	1	0
A. judaica	0.6	0	0	1

Table S7 (Continued)

Primer OPA-09

	A. annua	*A. herba-alba*	*A. monosperma*	*A. judaica*
A. annua	1	0	0	0.1667
A. herba-alba	0	1	0.3333	0.3333
A monosperma	0	0.3333	1	0.2
A. judaica	0.1667	0.3333	0.2	1

Primer OPD-15

	A. annua	*A. herba-alba*	*A. monosperma*	*A. judaica*
A. annua	1	0.1667	0.1667	0
A. herba-alba	0.1667	1	1	0
A monosperma	0.1667	1	1	0
A. judaica	0	0	0	1

Primer OPB-19

	A. Annua	*A. herba-alba*	*A. monosperma*	*A. judaica*
A. annua	1	0.1428	0	0.2857
A. herba-alba	0.1428	1	0	0.75
A monosperma	0	0	0	0
A. judaica	0.2857	0.75	0	1

Primer OPAT-19

	A. annua	*A. herba-alba*	*A. monosperma*	*A. judaica*
A. annua	1	0	0	0
A. herba-alba	0	0	0	0
A monosperma	0	0	1	0
A. judaica	0	0	0	1

Primer OPK-07

	A. Annua	*A. herba-alba*	*A. monosperma*	*A. judaica*
A. annua	1	0	0	0
A. herba-alba	0	1	0	0
A monosperma	0	0	1	0
A. judaica	0	0	0	1

Primer OPS-12

	A. annua	*A. herba-alba*	*A. monosperma*	*A. judaica*
A. annua	1	0	1	0
A. herba-alba	0	1	0	0.5
A monosperma	1	0	1	0
A. judaica	0	0.5	0	1

Primer OPB-13

	A. annua	*A. herba-alba*	*A. monosperma*	*A. judaica*
A. annua	1	0	0.25	0.5
A. herba-alba	0	1	0	0
A monosperma	0.25	0	1	0.5
A. judaica	0.5	0	0.5	1

Primer OPA-04

	A. annua	*A. herba-alba*	*A. monosperma*	*A. judaica*
A. annua	1	0.6	0.1666	0.4285
A. herba-alba	0.6	1	01666	0.25
A monosperma	0.1666	0.1666	1	0.2857
A. judaica	0.4285	0.25	0.2857	1

Primer OPB-17

	A. Annua	*A. herba-alba*	*A. monosperma*	*A. judaica*
A. annua	1	0	0	0.2
A. herba-alba	0	1	0.6667	0
A monosperma	0	0.6667	1	0
A. judaica	0.2	0	0	1

Primer OPB-07

	A. qnnua	*A. herba-alba*	*A. monosperma*	*A. judaica*
A. annua	1	0	0	0
A. herba-alba	0	1	0	0.25
A monosperma	0	0	1	0
A. judaica	0	0.25	0	1

Table S7 (Continued)

Primer OPA-16

	A. Annua	*A. herba-alba*	*A. monosperma*	*A. judaica*
A. annua	1	0.1111	0.1666	0.2857
A. herba-alba	0.1111	1	0.2857	0.2222
A. monosperma	0.1666	0.2857	1	0.3333
A. judaica	0.2857	0.2222	0.3333	1

Primer OPB-03

	A. annua	*A. herba-alba*	*A. monosperma*	*A. judaica*
A. annua	1	0.5	0	0
A. herba-alba	0.5	1	0.5	0
A monosperma	0	0.5	1	0
A. judaica	0	0	0	1

Primer OPB-20

	A. annua	*A. herba-alba*	*A. monosperma*	*A. judaica*
A. annua	1	0	0	0
A. herba-alba	0	1	0	1
A monosperma	0	0	1	0
A. judaica	0	1	0	1

Primer OPAH-17

	A. annua	*A. herba-alba*	*A. monosperma*	*A. judaica*
A. annua	1	0	0.5	0
A. herba-alba	0	1	0	0.75
A monosperma	0.5	0	1	0
A. judaica	0	0.75	0	1

Primer OPA-02

	A. Annua	*A. herba-alba*	*A. monosperma*	*A. judaica*
A. annua	1	0.5	0	0
A. herba-alba	0.5	1	0	0.1666
A monosperma	0	0	1	0.2
A. judaica	0	0.1666	0.2	1

Primer OPA-14

	A. annua	*A. herba-alba*	*A. monosperma*	*A. judaica*
A. annua	0	0	0	0
A. herba-alba	0	1	0	0
A monosperma	0	0	0	0
A. judaica	0	0	0	1

Primer OPB-18

	A. Annua	*A. herba-alba*	*A. monosperma*	*A. Judaica*
A. annua	1	0.1428	0	0
A. herba-alba	0.1428	1	0	0
A monosperma	0	0	1	0
A. judaica	0	0	0	1

Table S8 UPGMA trees of the studied *Artemisia* species based on Jaccard similarity coefficient of different RAPD primers.

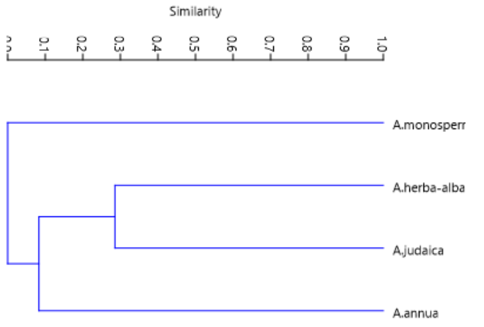

Primer OPG-02

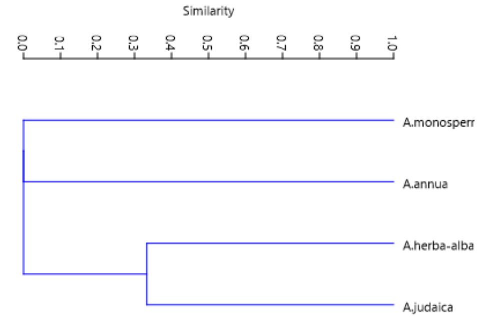

Primer OPG-03

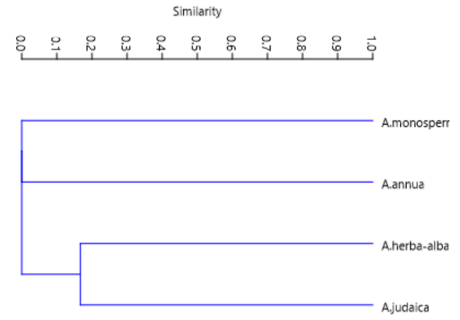

Primer OPG-04

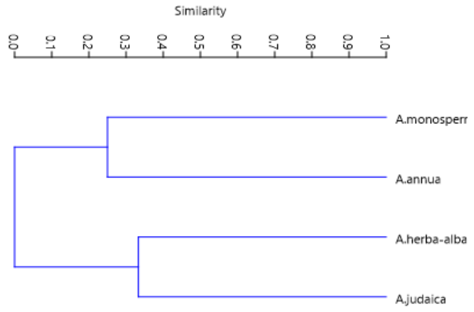

Primer OPG-05

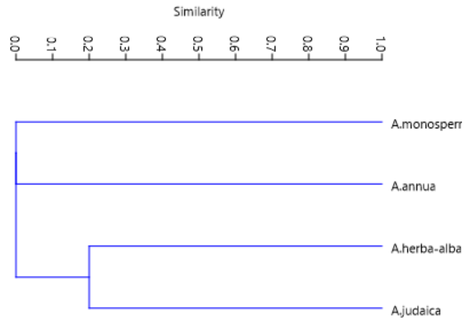

Primer OPG-06

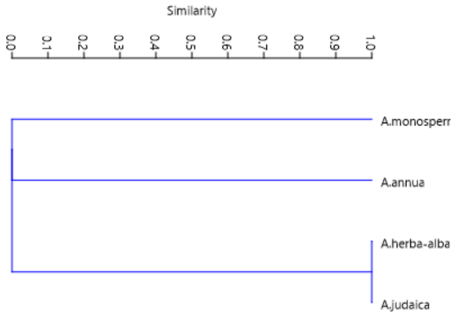

Primer OPG-07

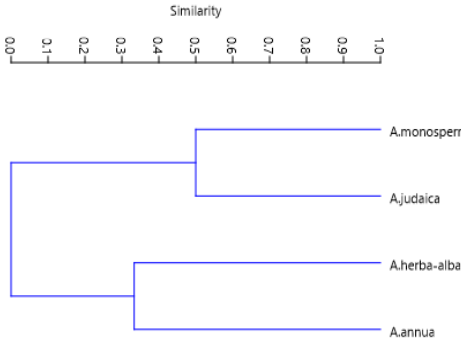

Primer OPG-08

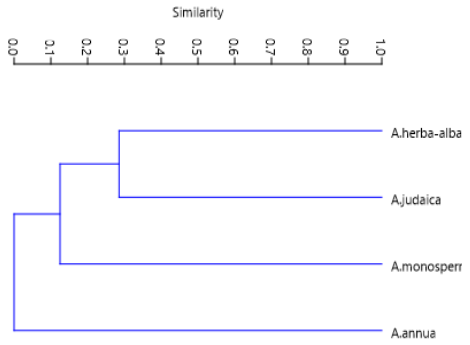

Primer OPG-09

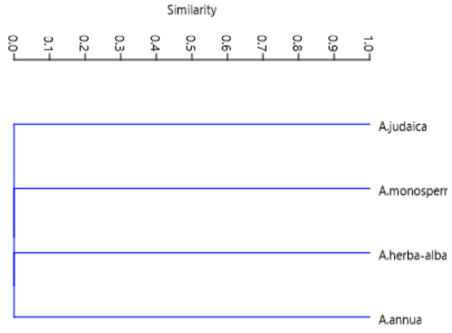

Primer OPA-10

Table S8 (Continued)

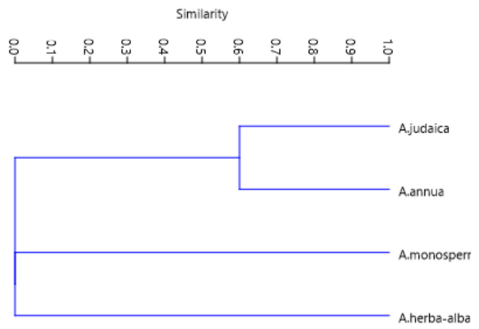

Primer OPA-11

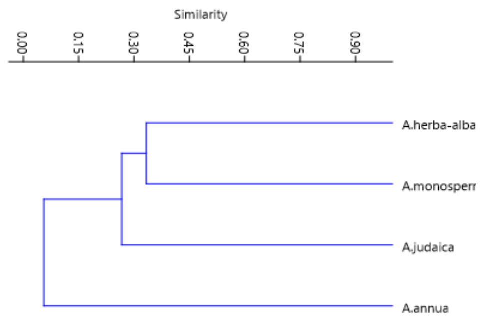

Primer OPA-09

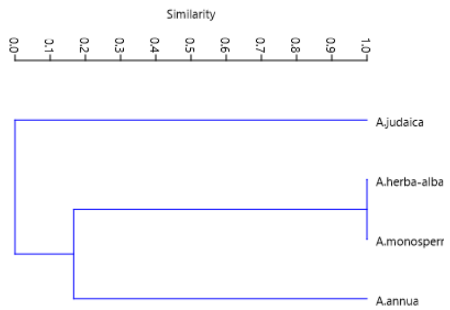

Primer OPD-15

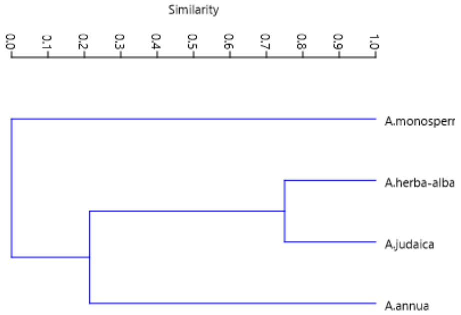

Primer OPB-19

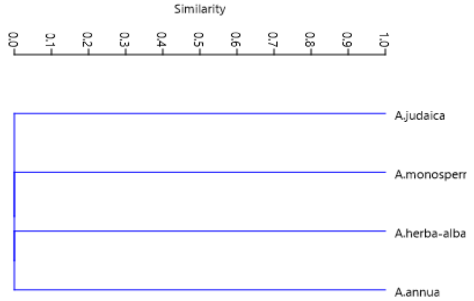

Primer OPAT-19

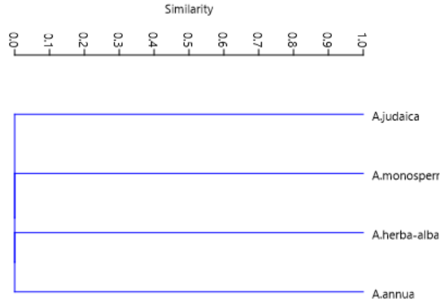

Primer OPK-07

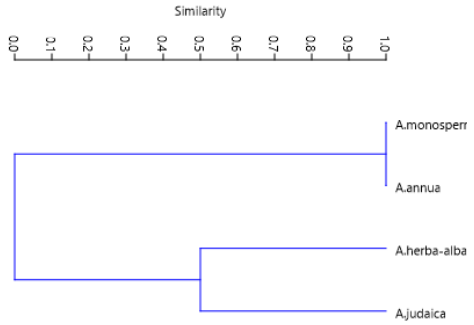

Primer OPS-12

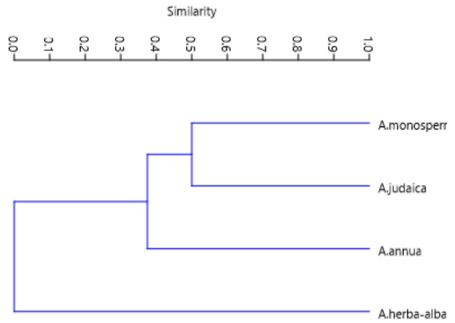

Primer OPB-13

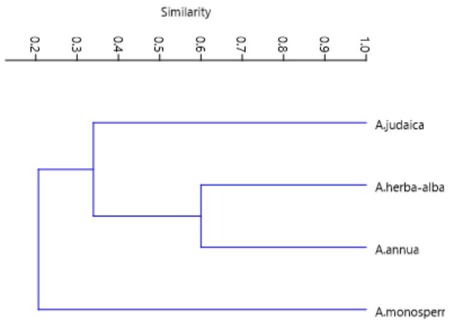

Primer OPA-04

Table S8 (Continued)

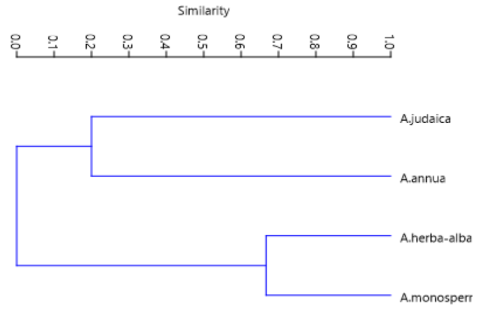

Primer OPB-17

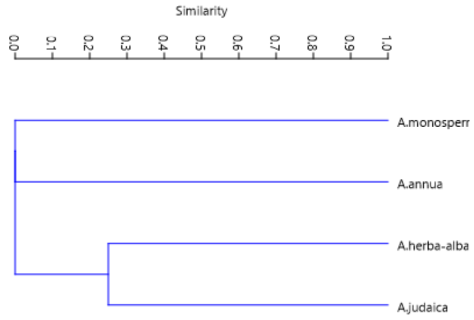

Primer OPB-07

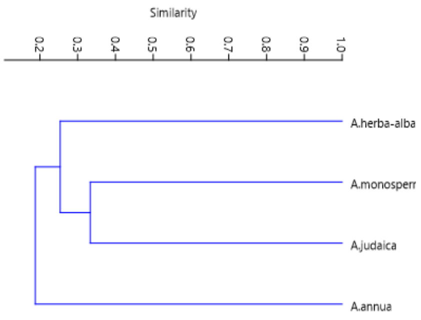

Primer OPA-16

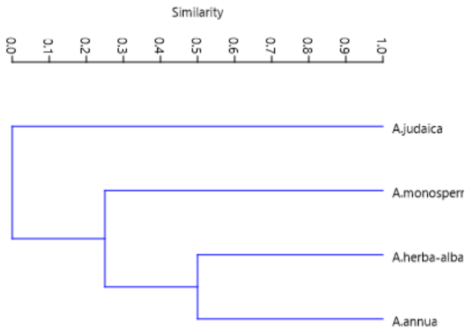

Primer OPB-03

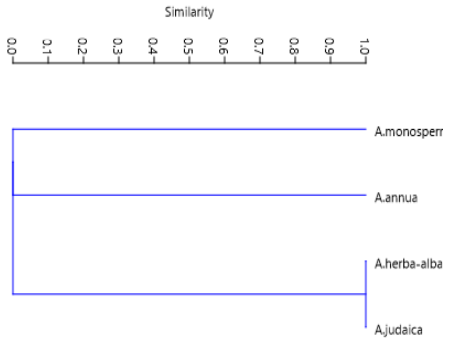

Primer OPB-20

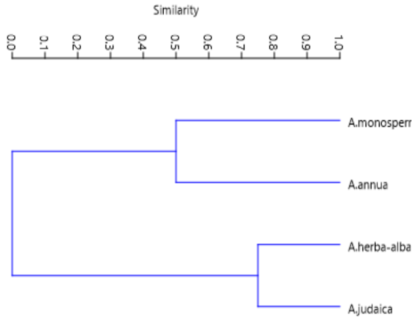

Primer OPAH-17

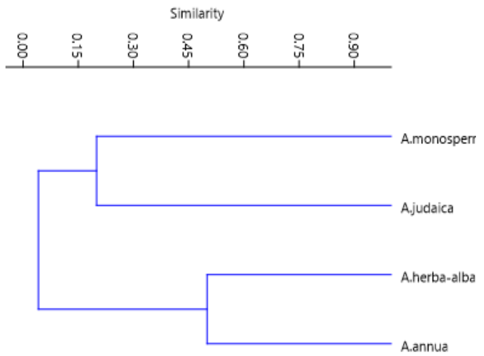

Primer OPA-02

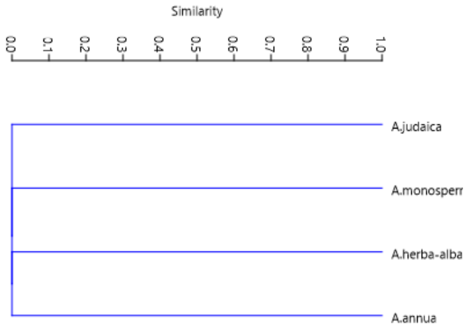

Primer OPA-14

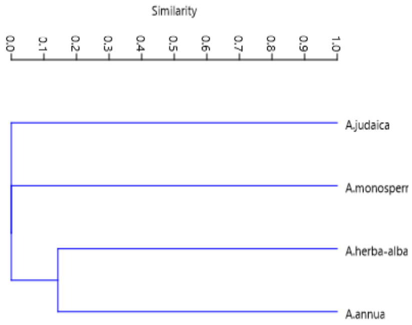

Primer OPB-18

Table S9 Collective Jaccard 's similarity coefficient of the 27 selected RAPD primers.

	A. annua A.A1 2023	***A. herba-alba*** A.H1 2023	***A. monosperma*** A.M1 2023	***A. judaica*** A.J1 2023
A. annua A.A1 2023	1	0.0869	0.0582	0.0851
A. herba-alba A.H1 2023	0.0869	1	0.1026	0.2098
A. monosperma A.M1 2023	0.0582	0.1026	1	0.0645
A. judaica A.J1 2023	0.0851	0.2098	0.0645	1

Table S10 Diversity parameters exhibited among different populations of the studied *Artemisia* species based on RAPD profiles.

**Plants**	**Na**	**Ne**	**H**	**I**	**PL**	**PPL**
A. annua	1.3019 (0.4602)	1.2415 (0.3681)	0.1342 (0.0204)	0.1922 (0.0293)	64	30.19%
A. herba-alba	1.3302 (0.4714)	1.2642 (0.3771)	0.1468 (0.0209)	0.2102 (0.03)	70	33.02%
A.monosperma	1.3160 (0.4660)	1.2528 (0.3728)	0.1405 (0.0207)	0.2012 (0.0297)	67	31.60%
A. judaica	1.3396 (0.4747)	1.2717 (0.3798)	0.1509 (0.0211)	0.2162 (0.0302)	72	33.96%

Observed number of alleles (Na), Effective number of alleles (Ne), Nei's gene diversity index (H), Shannon's information index(I), Number of polymorphic loci (PL) and Percentage of polymorphic loci (PPL) between populations and between species.

Table S11 The studied *Artemisia* species SCAR markers' sizes and accession numbers.

**Plant**	**Amplicon size (bp)**	**Accession number**
A.annua	223 bp	PV568346
A.herba-alba	440 bp	PV568347
A.monosperma	361 bp	PV568348
A.judaica	303 bp	PV568349

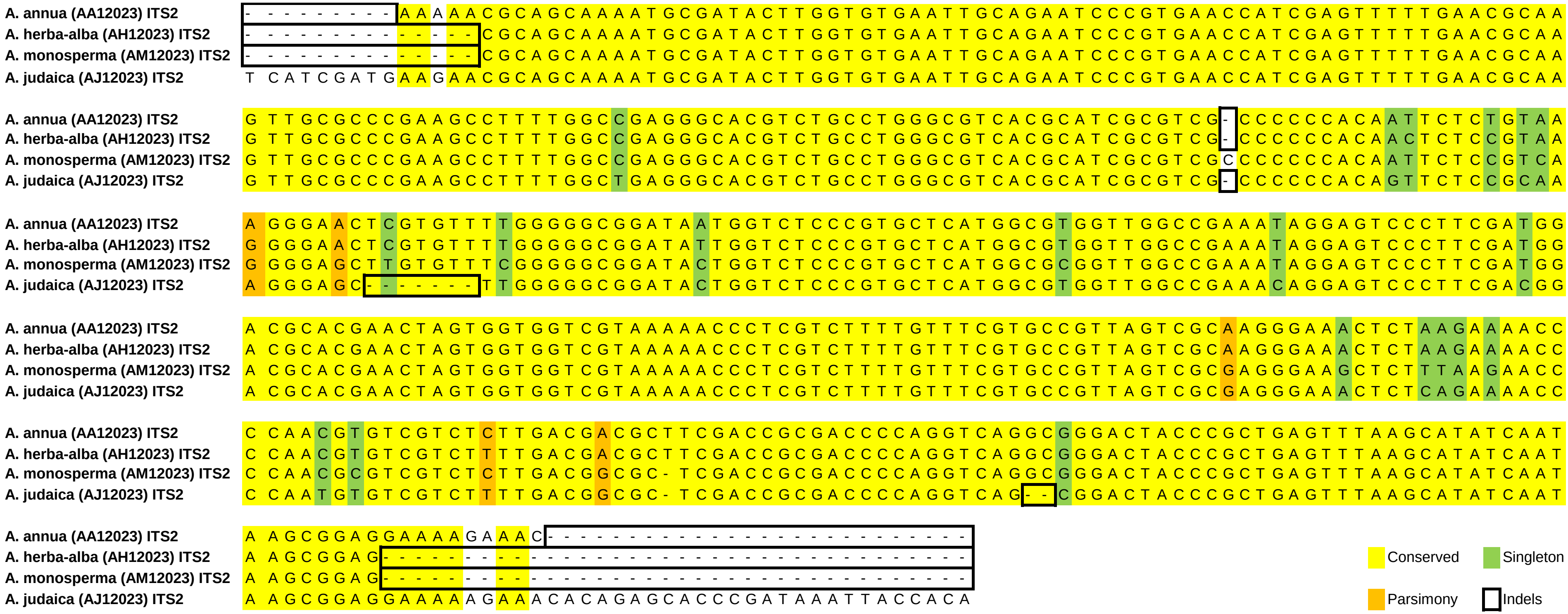

Fig. S1 Sequence alignment of representative ITS2 sequences of the studied *Artemisia* species.

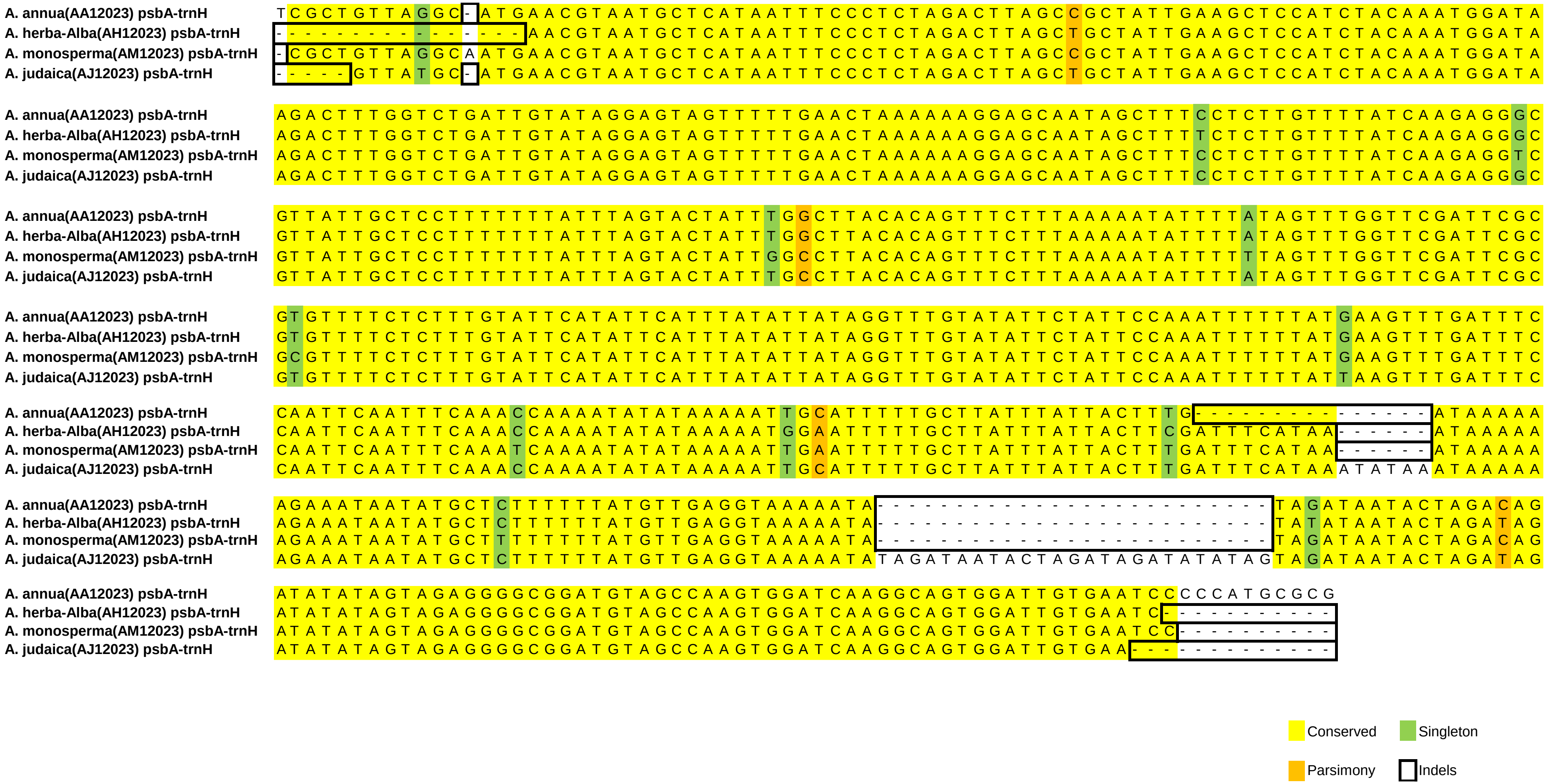

Fig. S2 Sequence alignment of representative psbA-trnH sequences of *Artemisia* species.

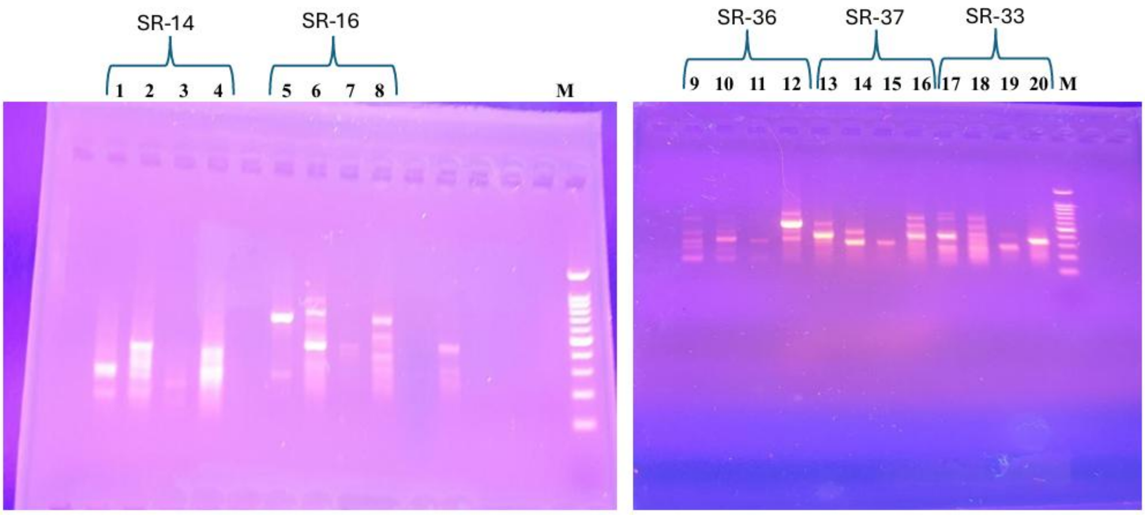

Fig. S3 Original uncropped gels of ISSR profiles of the studied Artemisia species using 5 ISSR primers; SR-14, SR-16, SR-36, SR-37 and SR-33, loaded against 100 bp DNA ladder (lane M). A. annua was loaded in (lanes 1, 5, 9, 13 and 17), A. herba-alba was loaded in (lanes 2, 6, 10, 14 and 18), A. monosperma was represented by (lanes 3, 7, 11, 15 and 19) and A. judaica by (lanes 4, 8, 12, 16 and 20).

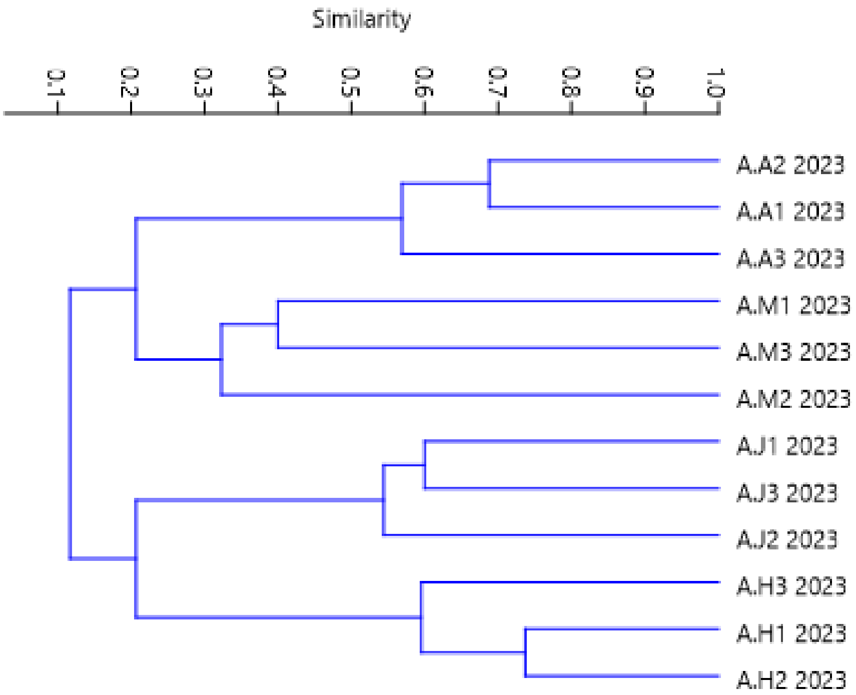

Fig. S4 UPGMA tree illustrating the genetic similarity among the examined populations of the studied *Artemisia* species based on the collective ISSR fingerprinting using the five selected ISSR primers.

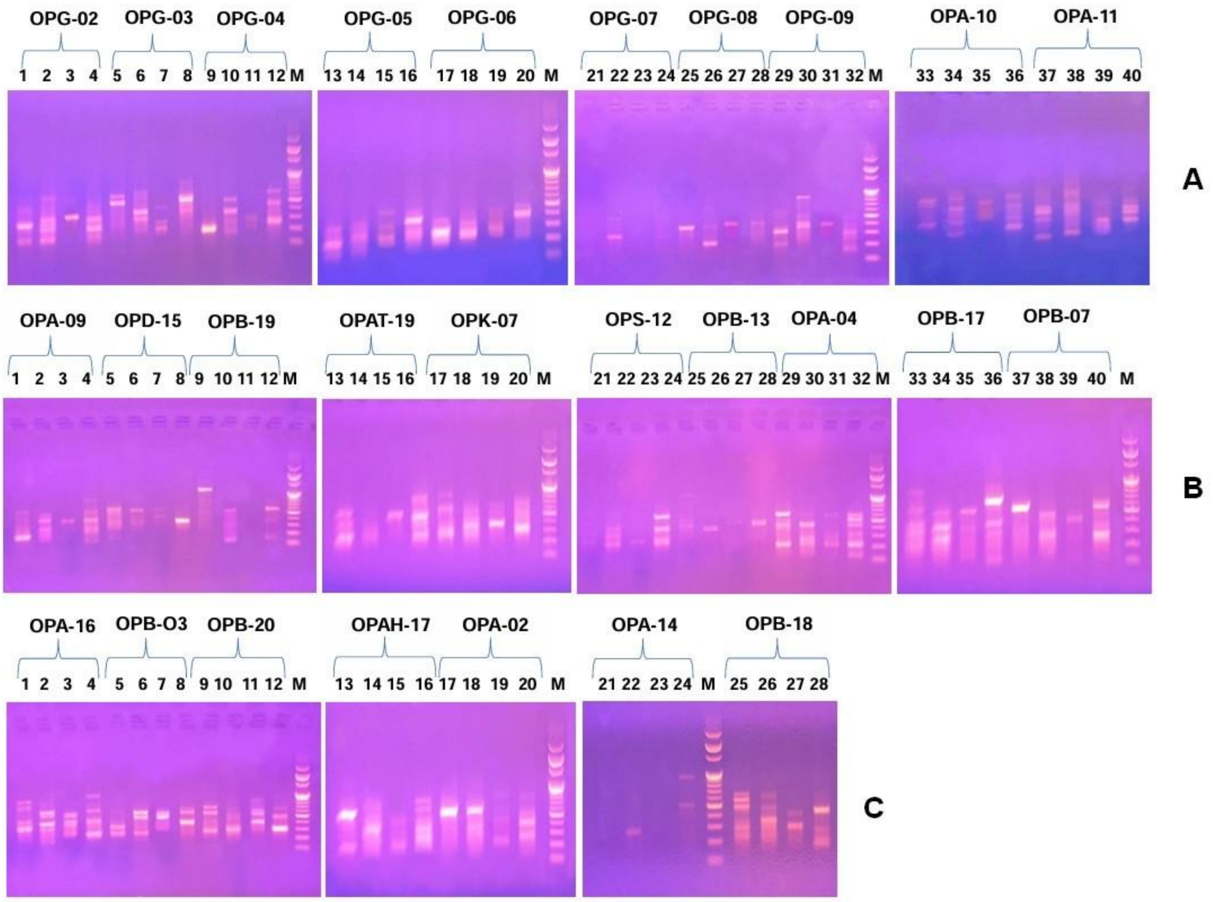

Fig. S5 RAPD profiles generated for the studied *Artemisia* species. *A. annua* (lanes 1, 5, 9, 13, 17, 21, 25, 29, 33 and 37), *A. herba-alba* (lanes 2, 6, 10, 14, 18, 22, 26, 30, 34 and 38) *, A. monosperma* (lanes 3,7, 11, 15, 19, 23, 27, 31, 35 and 39) *and A. judaica* (lanes 4, 8, 12, 16, 20, 24, 28, 32, 36 and 40) using RAPD primers; **(A)** OPG-02, OPG-03, OPG-04, OPG-05, OPG-06, OPG-07, OPG-08, OPG-09, OPA-10 and OPA-11, **(B)** OPA-09, OPD-15, OPB-19, OPAT-19, OPK-07, OPS-12, OPB-13, OPA-04, OPB-17 and OPB-07, **(C)** OPA-16, OPB-03, OPB-20, OPAH-17, OPA-02, OPA-14 and OPB-18, respectively along with 100 bp DNA ladder (lane M). Original uncropped gels are presented in Fig. S6.

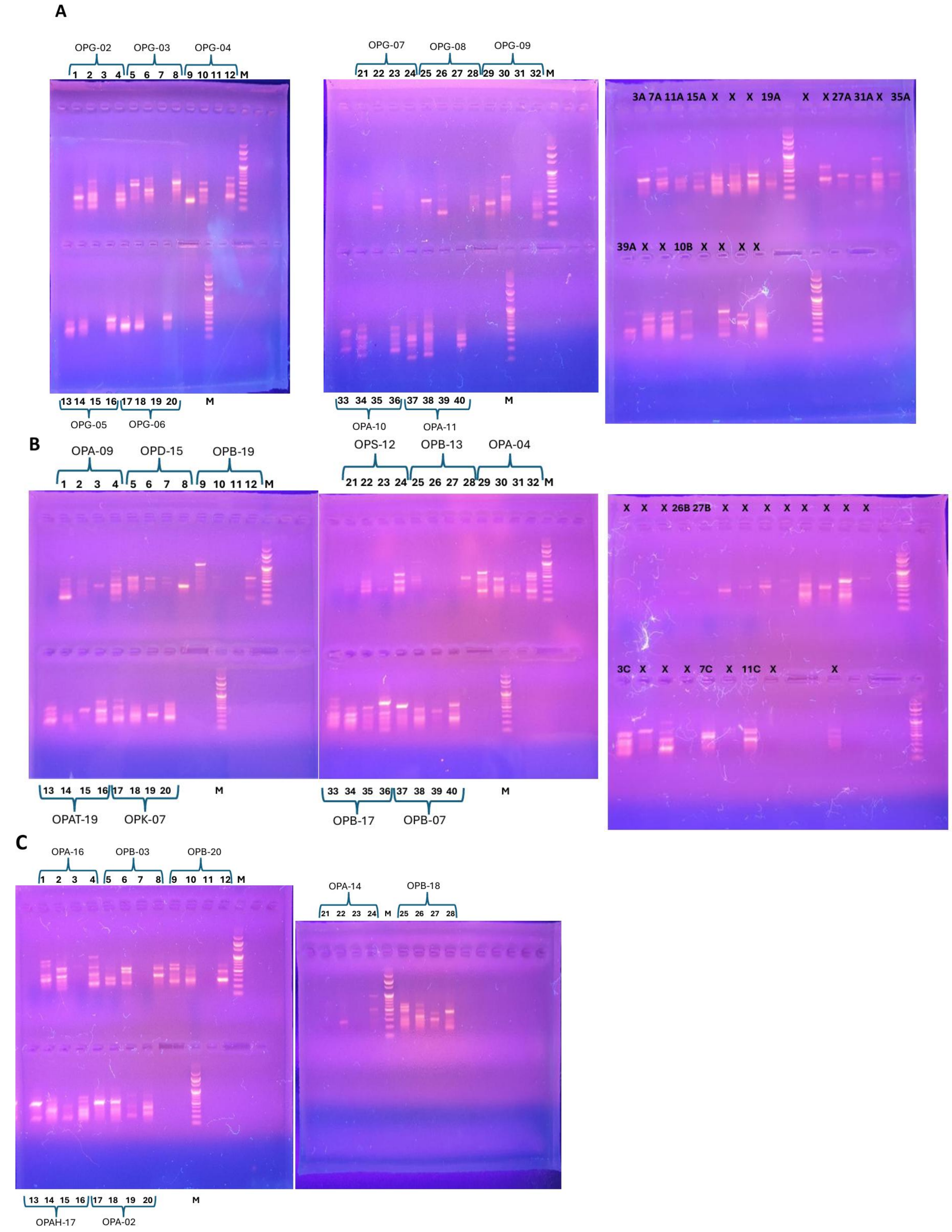

Fig. S6 Original uncropped gels of RAPD profiles generated for the studied *Artemisia* species. *A. annua* (lanes 1, 5, 9, 13, 17, 21, 25, 29, 33 and 37), *A. herba-alba* (lanes 2, 6, 10, 14, 18, 22, 26, 30, 34 and 38) , *A. monosperma* (lanes 3,7, 11, 15, 19, 23, 27, 31, 35 and 39) *and A. judaica* (lanes 4, 8, 12, 16, 20, 24, 28, 32, 36 and 40) using RAPD primers; **(A)** OPG-02, OPG-03, OPG-04, OPG-05, OPG-06, OPG-07, OPG-08, OPG-09, OPA-10 and OPA-11, **(B)** OPA-09, OPD-15, OPB-19, OPAT-19, OPK-07, OPS-12, OPB-13, OPA-04, OPB-17 and OPB-07, **(C)** OPA-16, OPB-03, OPB-20, OPAH-17, OPA-02, OPA-14 and OPB-18, respectively along with 100 bp DNA ladder (lane M).

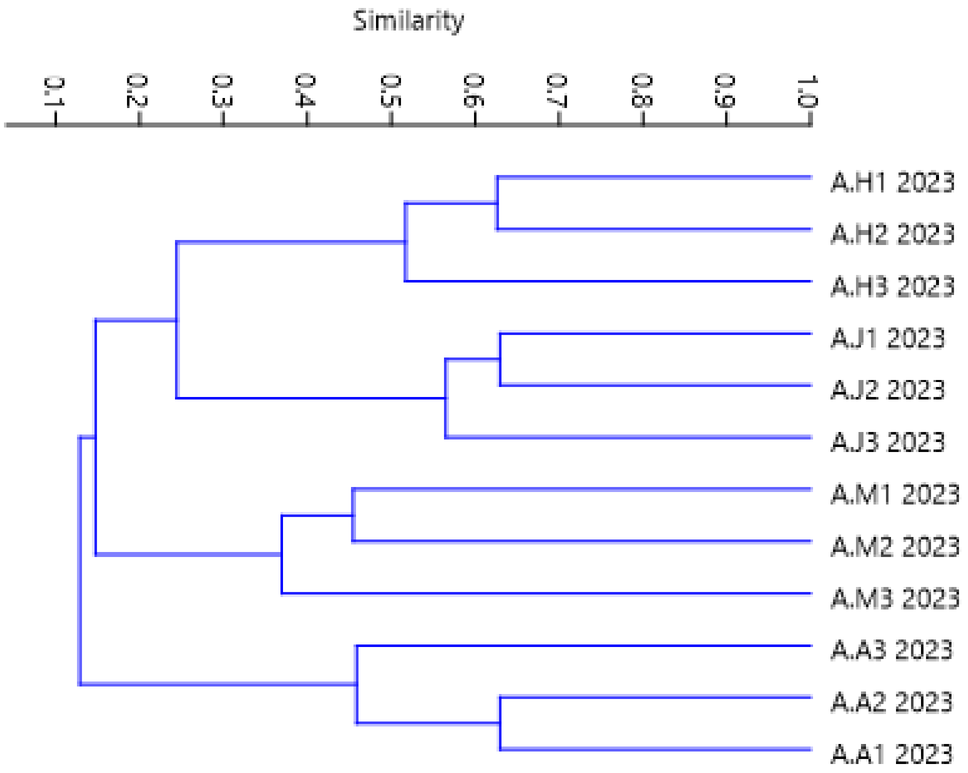

Fig. S7 UPGMA tree illustrating the genetic similarity between the examined populations of the studied *Artemisia* species based on the collective RAPD fingerprinting using the 27 selected RAPD primers.

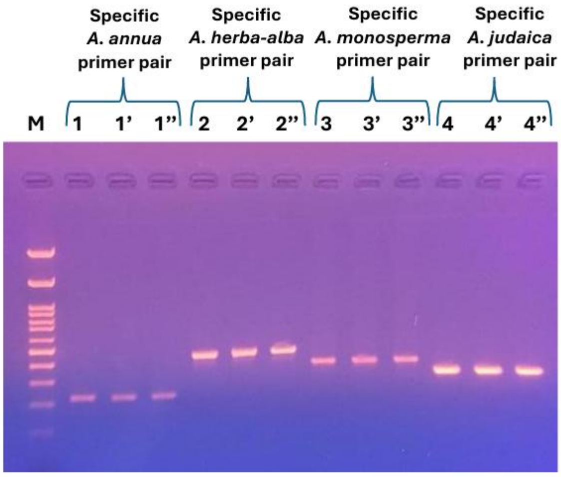

Fig. S8 The resultant RAPD based SCAR markers of samples of *A. annua* (Lanes 1,1' and 1”), *A. herba-alba* (lanes 2,2' and 2”), *A. monosperma* (lanes 3, 3’and 3”) and *A. judaica* (lanes 4, 4’ and 4”) using the designed specific primer pairs, loaded against 100 bp DNA ladder (lane M). Original uncropped gel is presented in Fig. S9.

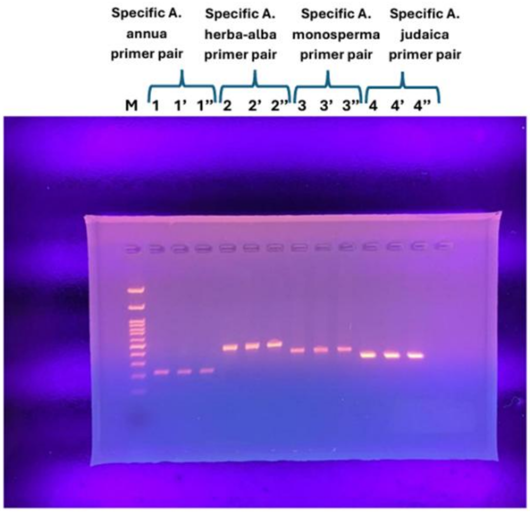

Fig. S9 Uncropped gel of the resultant RAPD based SCAR markers of samples of *A. annua* (Lanes 1,1' and 1”), *A. herba-alba* (lanes 2,2' and 2”), *A. monosperma* (lanes 3, 3’and 3”) and *A. judaica* (lanes 4, 4’ and 4”) using the designed specific primer pairs, loaded against 100 bp DNA ladder (lane M).

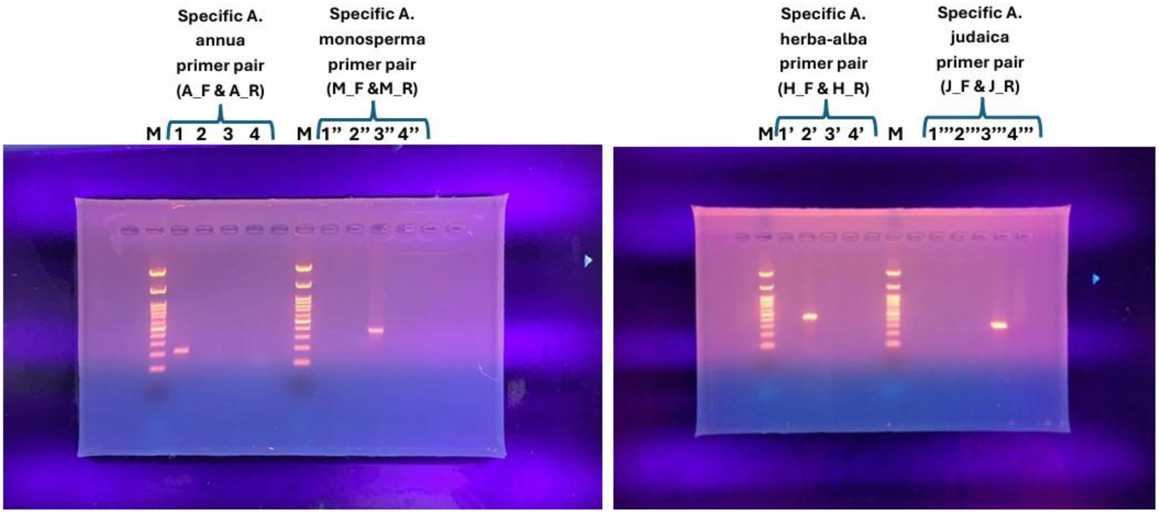

Fig. S10 Original uncropped gels of the resultant RAPD based SCAR markers for samples of A. annua (Lanes 1,1', 1'' and 1''') , A. herba-alba (lanes 2, 2', 2'' and 2'''), A. monosperma (lanes 3, 3', 3''and 3''') and A. judaica (lanes 4, 4', 4'' and 4''') using the designed specific primer pairs, loaded against 100 bp DNA ladder (lane M).
